# Environmental cues received during development shape dendritic cell responses later in life

**DOI:** 10.1371/journal.pone.0207007

**Published:** 2018-11-09

**Authors:** Jessica L. Meyers, Bethany Winans, Erin Kelsaw, Aditi Murthy, Scott Gerber, B. Paige Lawrence

**Affiliations:** 1 Department of Environmental Medicine, University of Rochester School of Medicine & Dentistry, Rochester, New York, United States of America; 2 Department of Microbiology and Immunology, University of Rochester School of Medicine & Dentistry, Rochester, New York, United States of America; 3 Department of Surgery, University of Rochester School of Medicine & Dentistry, Rochester, New York, United States of America; University of South Carolina School of Medicine, UNITED STATES

## Abstract

Environmental signals mediated via the aryl hydrocarbon receptor (AHR) shape the developing immune system and influence immune function. Developmental exposure to AHR binding chemicals causes persistent changes in CD4^+^ and CD8^+^ T cell responses later in life, including dampened clonal expansion and differentiation during influenza A virus (IAV) infection. Naïve T cells require activation by dendritic cells (DCs), and AHR ligands modulate the function of DCs from adult organisms. Yet, the consequences of developmental AHR activation by exogenous ligands on DCs later in life has not been examined. We report here that early life activation of AHR durably reduces the ability of DC to activate naïve IAV-specific CD8^+^ T cells; however, activation of naïve CD4^+^ T cells was not impaired. Also, DCs from developmentally exposed offspring migrated more poorly than DCs from control dams in both *in vivo* and *ex vivo* assessments of DC migration. Conditional knockout mice, which lack *Ahr* in CD11c lineage cells, suggest that dampened DC emigration is intrinsic to DCs. Yet, levels of chemokine receptor 7 (CCR7), a key regulator of DC trafficking, were generally unaffected. Gene expression analyses reveal changes in *Lrp1*, *Itgam*, and *Fcgr1* expression, and point to alterations in genes that regulate DC migration and antigen processing and presentation as being among pathways disrupted by inappropriate AHR signaling during development. These studies establish that AHR activation during development causes long-lasting changes to DCs, and provide new information regarding how early life environmental cues shape immune function later in life.

## Introduction

The immune system develops during gestation and following birth. The environment experienced *in utero* and during early postnatal life can influence the immune system, leading to durable changes that influence health later in life. For instance, reports from human population studies show that prenatal and early postnatal exposure to certain pollutants correlates with immune dysregulation later in life [1–4]. Research in animal models further supports the idea that immune function at maturity is influenced by early life exposures [5–9], and that the developing immune system is more sensitive than the mature immune system to enduring modulation by environmental factors [10–12]. While these studies reveal links between developmental exposures and life-long changes in immune function, how early life exposures shape the immune system is not fully understood.

Recent reports show that activation of the aryl hydrocarbon receptor (AHR) during development changes immune responses later in life, suggesting that this environment-sensing transcriptional regulator is one means via which the early life environment influences immune function. The AHR binds many synthetic and naturally derived chemicals that are commonly found in environment, including pollutants, dietary substances, and byproducts of microorganisms [13, 14]. The AHR is widely expressed in most tissues and cells, including immune cells, and is emerging as a key regulator of immune cell development, differentiation, and function [15, 16]. One group of exogenous AHR ligands to which humans are regularly exposed are dioxins and polychlorinated biphenyls (PCBs). These anthropogenic chemicals bioaccumulate in the food chain, and exposure is primarily via the diet [17, 18]. Fetuses and neonates are exposed as well, as dioxins and PCBs cross the placenta, and are found in breast milk, cord, and infant blood [19–22]. Moreover, several different human cohort studies show strong associations between early life exposure to these AHR-binding pollutants and altered immune function, including more severe or frequent respiratory infections and decreased antibody responses to vaccinations [23–28]. In mice, early life exposure to a prototype environmental AHR ligand, 2,3,7,8-tetrachlorodibenzo-*p*-dioxin (TCDD), causes durable alterations in T cell responses later in life. For example, developmentally exposed adult offspring have significantly fewer virus-specific CD8^+^ cytotoxic T lymphocytes (CTL) and conventional CD4^+^ T helper cells after infection with influenza A virus [7, 8, 29, 30].

In order for naïve T cells to become activated, proliferate, and differentiate into appropriately armed effector cells, they must receive an integrated set of signals from dendritic cells (DCs). The AHR is emerging as an important regulator of DC function, although this evidence is from studies of direct exposure (i.e., not developmental exposure) to AHR ligands [31–39]. However, whether triggering of the AHR during development changes the function of DCs later in life has not been investigated. In the present study, we determined whether maternal exposure to a representative environmental AHR ligand modifies DC function in adult offspring, including their ability to stimulate naïve T cells, their frequency and distribution *in vivo*, and their migration. We used targeted transcriptome analyses to investigate intrinsic differences in gene expression in DCs from developmentally exposed mice. Our results show that AHR activation during development induces long-lasting changes in DC functions. Given the pivotal role that DCs play in establishing and maintaining T cell responses, the ability of early life AHR activation to affect DC functional properties later in life has many implications as we seek to understand AHR-mediated regulation of the immune system, and to understand how the early life environmental cues shape the way the immune system is poised to respond.

## Results

### AHR activation during development reduces the ability of DCs to stimulate naïve CD8^+^ T cells, but not naïve CD4^+^ T cells

**Fig 1:**
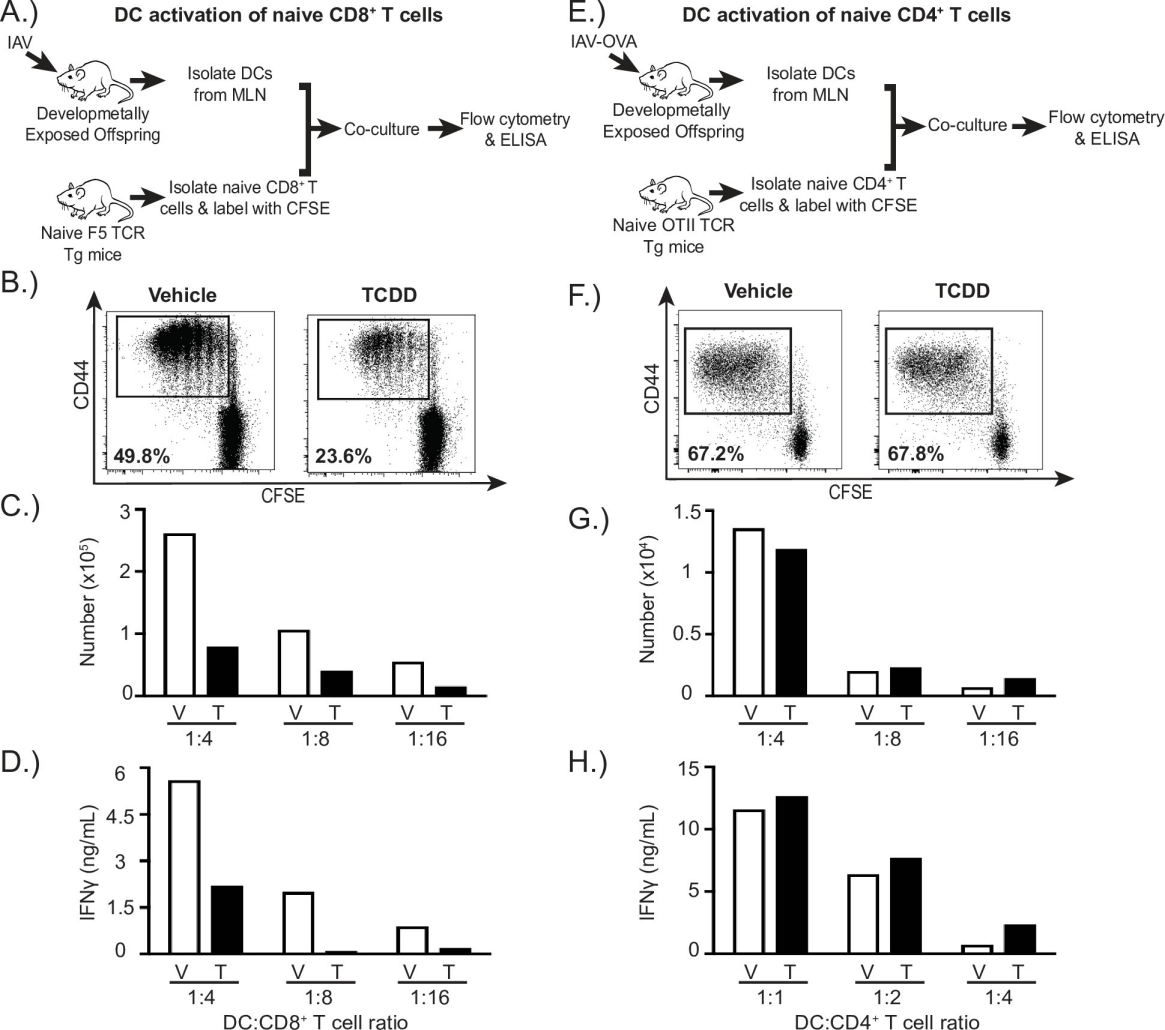
DCs from developmentally exposed mice have an impaired ability to stimulate naïve virus-specific CD8^+^ T cells but not naïve CD4^+^ T cells. **(A)** Adult offspring of dams treated with vehicle (V) or TCDD (T) were infected (i.n.) with IAV (Memphis/102/72). On day 3 post infection, MLNs were pooled from animals of the same group (≥ 30 mice/group), and DCs enriched with immunomagnetic separation. Naïve (CD44^lo^) CD8^+^ T cells were isolated from spleens of untreated and uninfected F5 TCR transgenic mice, and labeled *ex vivo* with CFSE. Serially diluted DCs were and co-cultured with CFSE-labeled naïve F5 CD8^+^ T cells (2x10^5^ T cells/well) in a range from 1:4–1:16 DCs:T cells. After 3 days in culture, cells were collected and stained for flow cytometric analysis. (**B**) The dot plots indicate the percentage of proliferating (CD44^hi^CFSE^decay^) F5 CD8^+^ T cells after culture with DCs from vehicle or TCDD exposed offspring (1:4 DC:T cell ratio). F5 CD8^+^ cells were identified as Vβ11^+^CD8^+^ cells. **(C)** The bar graph shows the number of proliferating F5 CD8^+^ T cells. (**D**) The graph depicts IFNγ levels in supernatants at diminishing DC:T cell ratios. (**E**) Adult offspring were infected (i.n.) with HKx31/OVAII (≥ 30 mice/group). On day 3 post infection, MLNs were pooled from animals of the same group (≥ 30 mice/group), and DCs were enriched from the pool of MLN cells. Naïve (CD44^lo^) CD4^+^ T cells were isolated from spleens of untreated and uninfected OTII TCR transgenic mice and labeled with CFSE. DCs were co-cultured with CFSE labeled naïve OTII CD4^+^ T cells (2x10^5^ T cells/well) for 4 days in a range from 1:4–1:16 DCs:T cells. (**F**) The dot plots indicate the percentage of proliferating (CD44^hi^CFSE^decay^) OTII CD4^+^ T cells after culture with DCs from vehicle or TCDD exposed offspring (1:4 DC:T cell ratio). OTII CD4^+^ cells were identified as Vβ5^+^CD4^+^ cells. **(G)** Bar graphs show the number of proliferating OTII CD4^+^ T cells stimulated by DCs. (**H**) Bar graph shows IFNγ levels in supernatant at indicated DC:T cell ratios. Data are from one representative experiment, except IFNγ levels in DC:OTII CD4^+^ T cell co-cultures, which show the combined data from two experiments. Each experiment was independently repeated at least 2 times with similar results. Underlying data can be found in S1 Data.

During a primary immune challenge, naïve T cells require activation signals from DCs. Given that developmental exposure to environmental AHR ligands, such as TCDD, durably disrupts CD8^+^ and CD4^+^ T cell responses [7, 8, 29, 40], we sought to determine whether early life AHR activation affects the ability of DCs to activate naïve T cells using an established assay of APC function [35, 38]. Specifically, after respiratory infection we isolated DCs from the lung-draining mediastinal lymph nodes (MLN), the principle site of naïve T cell activation during IAV infection. We used an equivalent number of DCs from adult infected offspring of vehicle or TCDD exposed dams to activate naïve, CFSE-labeled TCR transgenic T cells *ex vivo*. To measure activation of CD8^+^ T cells, DCs were isolated from the MLN of developmentally exposed, infected adult offspring and co-cultured with naïve CD8^+^ T cells from naïve, untreated F5 TCR transgenic mice (Fig 1A). The TCR on CD8^+^ T cells of F5 mice recognizes a specific immunodominant peptide from IAV nucleoprotein (NP_366-374_) [41]. Compared to DCs isolated from infected offspring of vehicle-treated dams, DCs from adult offspring of TCDD-treated dams had a two-fold reduction in the percentage of activated and proliferated (CD44^hi^CFSE^decay^) F5 CD8^+^ T cells (Fig 1B). The number of CD44^hi^CFSE^decay^CD8^+^ T cells was also reduced when F5 CD8^+^ T cells were cultured with DCs from offspring of TCDD treated dams (Fig 1C). To examine CD8^+^ T cell differentiation, we measured IFNγ levels. CD8^+^ T cells cultured with DCs from offspring of TCDD-treated dams secreted roughly half as much IFNγ as CD8^+^ T cells stimulated by DCs from vehicle controls (Fig 1D). These data indicate that AHR activation during development reduced the ability of DCs to act as optimally effective APCs for naïve CD8^+^ T cells, as they stimulated lower levels of CD8^+^ T cell proliferation and differentiation. That is, activation of the AHR early in life, via direct treatment of the dam and vertical exposure to the fetus and neonate, leads to perturbation in the ability of DCs to stimulate naïve CD8^+^ T cells in adult offspring.

CD4^+^ T cells are also important for host defenses against IAV and other respiratory pathogens [42]. Therefore, we investigated whether developmental exposure affected the ability of DCs to stimulate naïve CD4^+^ T cells. We used a similar approach to the *ex vivo* system with naïve CD8^+^ T cells, with two changes. We isolated CD4^+^ T cells from naïve, untreated OTII TCR transgenic mice, whose CD4^+^ T cells bear TCRs that recognize a peptide fragment of ovalbumin (OVA_323-339_), and isolated DCs from developmentally exposed mice infected with a transgenic IAV that expresses this peptide: x31/OVAII [43, 44]. After infection, DCs from MLN of developmentally exposed adult offspring were co-cultured with naïve CFSE-labeled naïve CD4^+^ T cells from OTII TCR transgenic mice (Fig 1E). Regardless of whether the offspring were from vehicle or TCDD treated dams, DCs stimulated a similar percentage and number of OTII CD4^+^ T cells to become activated and proliferate (Fig 1F and 1G). Furthermore, these CD4^+^ T cells produced similar levels of IFNγ (Fig 1H). Thus, in contrast to an impaired ability to stimulate CD8^+^ T cells, early life AHR activation does not appear to affect the ability of DCs to serve as APCs for naïve CD4^+^ T cells in the context of IAV infection.

### In vivo distribution of DCs

**Fig 2:**
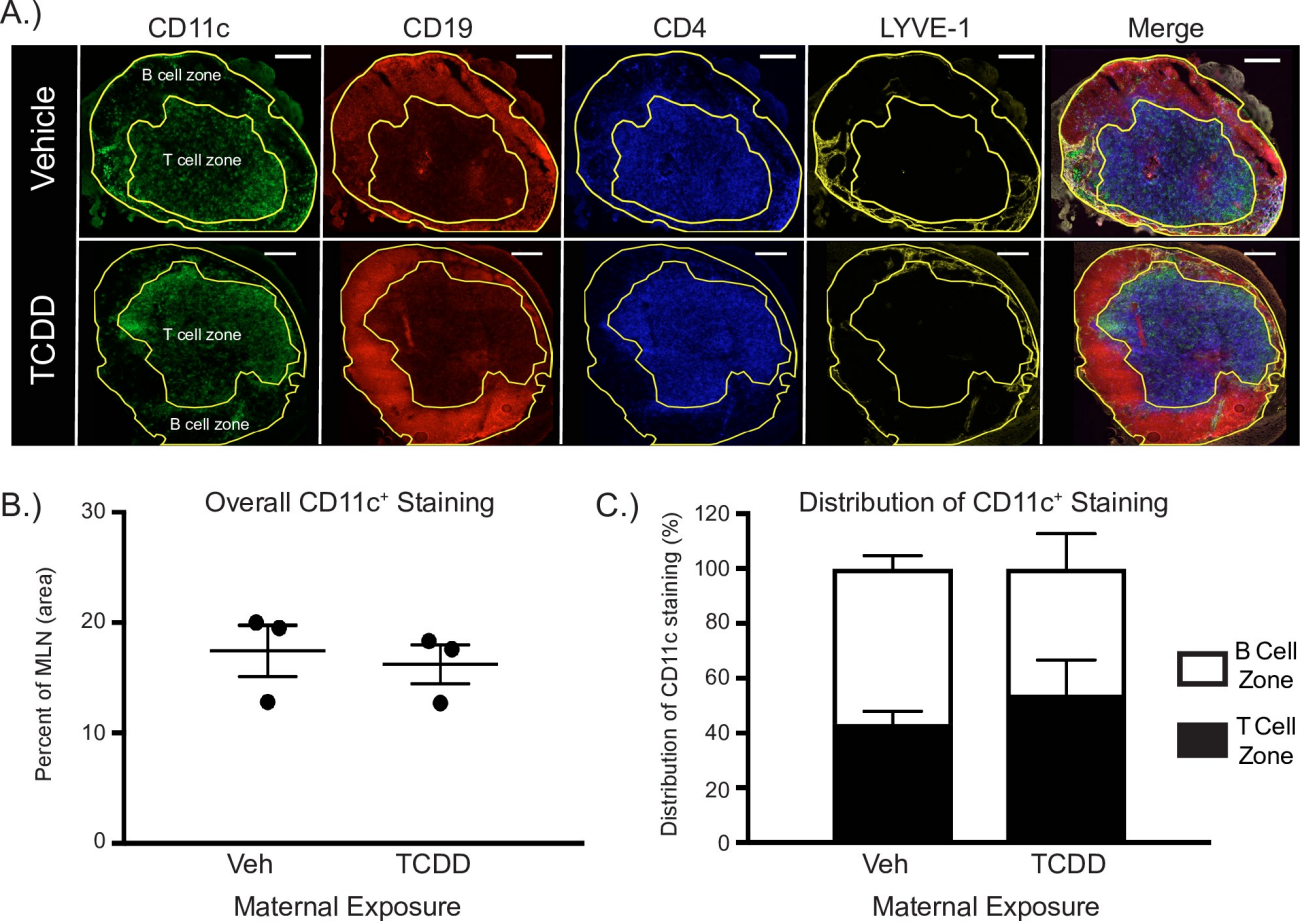
Developmental activation of the AHR does not alter the overall distribution of DCs in T or B cell zones in the MLN. (**A**) Representative fluorescent immunohistochemistry images from mature male offspring of dams exposed to vehicle (V; top row) or TCDD (T; bottom row) 3 days after infection with IAV (HKx31). Serial frozen sections were stained with anti-CD11c (DCs, green), anti-CD19 (B cells, red), anti-CD4 (CD4^+^ T cells, blue), and anti-LYVE-1 (lymphatic vessels, yellow) are shown, along with the merged image. The B cell zone and T cell zones were defined using CD19 or CD4, respectively. The outer boundary of the tissue section was defined using LYVE-1. Scale bar, 200 μm. (**B**) The graph shows the average percentage (± SEM) of CD11c staining throughout the MLN cross-sections analyzed. (**C**) The stacked bar graph shows the average percentage (± SEM) of positive CD11c staining in the B cell zone (white bars) and T cell zone (black bars) in MLN of vehicle (V) or TCDD (T) offspring. 3–8 sections analyzed/per mouse, from 3 different mice per group. Underlying data can be found in S1 Data.

In addition to affecting their ability to activate naive T cells, early life exposure could alter the distribution of DC within lymph nodes, which could contribute to poorer T cell responses during *in vivo* immune challenge. Therefore, we evaluated whether developmental exposure affects the *in vivo* distribution of DCs using immunohistochemistry and enumerated distinct DC subsets using analytical flow cytometry. One of the main sites where DCs act as APCs for naïve T cells is the T cell zone in lymph nodes. We utilized immunofluorescent microscopy to visualize the distribution of DCs in the MLN 3 days following IAV infection (Fig 2A). The percentage of the MLN cross-sectional area that was positive for CD11c^+^ staining was not different between adult offspring from vehicle or TCDD treated dams (Fig 2B). Moreover, the relative distribution of CD11c^+^ staining in T and B cell zones was equivalent between treatment groups (Fig 2C). Specifically, 43% and 54% of CD11c staining was in T cell zones of MLNs from vehicle and TCDD groups, respectively. These results suggest that, at this macro-level, developmental activation of AHR does not change the overall distribution of DCs between the T cell and B cell zones of the MLN during early stages of the response to IAV infection; a time of peak DC interaction with naïve T cells.

**Fig 3:**
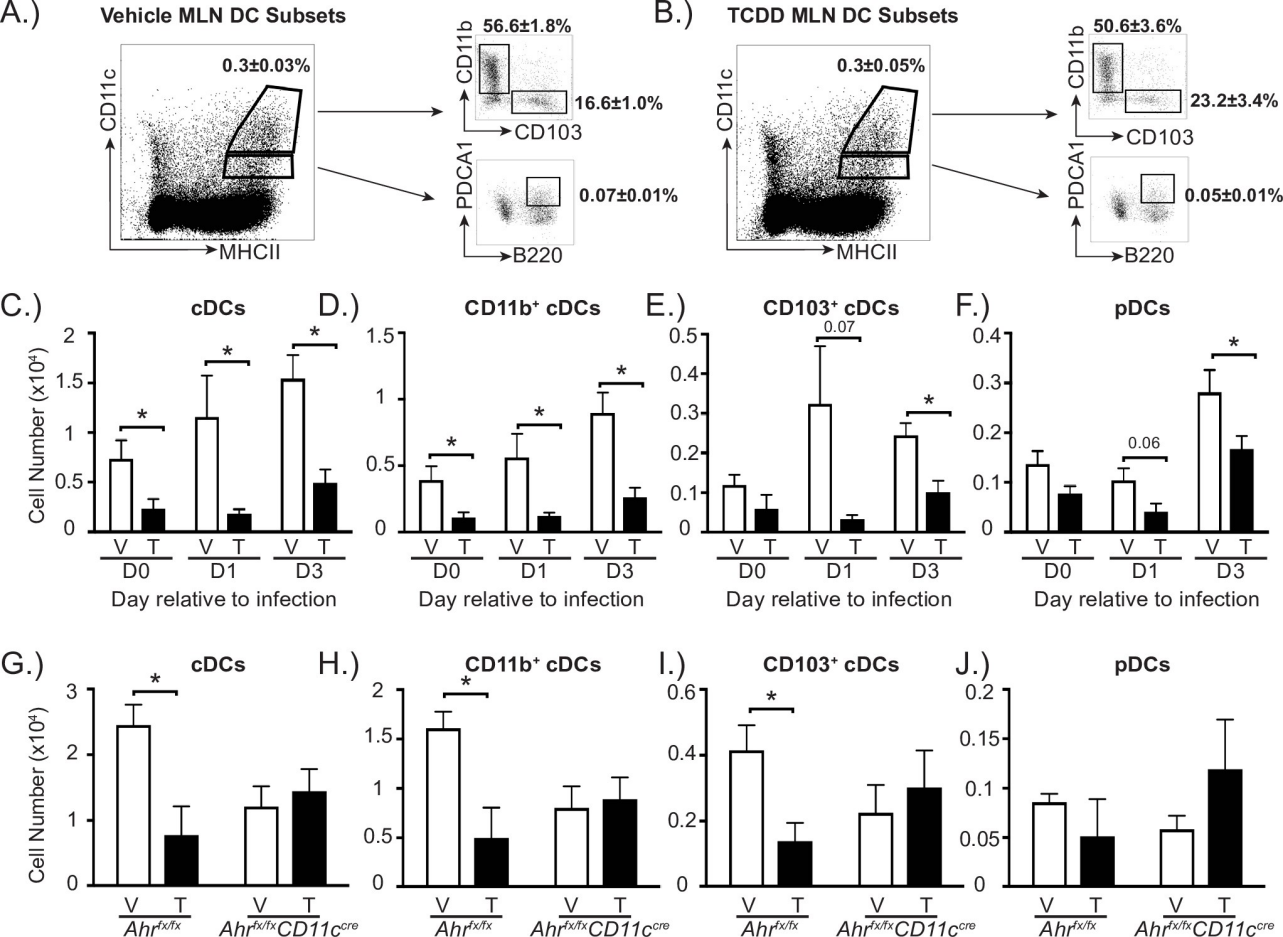
Activation of AHR during development reduces DCs in the MLN in an AHR-dependent manner. Mice were exposed to vehicle or TCDD during development. At maturity, developmentally exposed offspring were unchallenged, or infected with IAV (HKx31). The percentage and number of DC subsets in the MLN were determined by flow cytometry. **(A, B)** Following gating to exclude doublets and auto-fluorescent cells, DCs were identified as follows: conventional DCs (cDCs; CD11c^hi^MHCII^hi^ cells) and plasmacytoid DCs (pDCs; CD11c^lo^MHCII^hi^ PDCA1^+^CD45R^+^ cells). Conventional DCs were further subdivided into two populations: CD11b^+^ cDCs (CD11b^+^CD103^-^CD11c^hi^MHCII^hi^ cells) and CD103^+^ cDCs (CD103^+^CD11b^-^CD11c^hi^MHCII^hi^ cells). Representative dot plots depict the gating used to define cDCs, CD11b^+^ cDCs, CD103^+^ cDCs and pDCs in the MLN of vehicle (**A**) and TCDD (**B**) exposed offspring. The dot plots indicate the average percentage (±SEM) of the indicated DC subset 3 days after infection. **(C-F)** The bar graphs show the number (±SEM) of the indicated DC population in MLN from naïve (day 0) or infected mice. **(G-J)** Bar graphs show the number (±SEM) of DC in the MLN 3 days after IAV infection in *Ahr*^*fx/fx*^ or *Ahr*^*fx/fx*^*CD11c*^*cre*^ mice from V or T dams treated with 10 μg/kg BW TCDD on GD14 and PND2. At each point in time, all wildtype offspring within a group were from a separate dam, n = 6–9 mice per group per day. Some *Ahr*^*fx/fx*^ or *Ahr*^*fx/fx*^*CD11c*^*cre*^ mice offspring within a group were from the same dam, n = 3–5 mice per group, because of limited number of offspring. Day 0 data are representative of 4 independent experiments, day 1 data are representative of 3 independent experiments, day 3 data are representative of 6 independent experiments with similar results. The experiment using *Ahr*^*fx/fx*^*CD11c*^*cre*^ mice was performed once. An * indicates p ≤ 0.05. Underlying data can be found in S1 Data.

Activation of the AHR during development could also alter the number of distinct DC subsets within the MLN of the offspring. DCs are a heterogeneous population of cells, which are broadly categorized into conventional DCs (cDCs) and plasmacytoid DCs (pDCs) [45–47]. cDCs can be further divided into different subtypes. In the lung and lung-draining lymph nodes, two of the main types of cDCs are CD11b^+^ and CD103^+^ DCs. Both of these cDC subsets emigrate from the lung during respiratory antigen challenge, and present antigen to naïve T cells in the lung-draining lymph nodes [35, 38, 48–51]. We used multiple parameter analytical flow cytometry to identify and enumerate pDCs, total cDCs, CD11b^+^ cDCs and CD103^+^ cDCs, in adult offspring before and during IAV infection. Representative FACS plots depict the gating strategy to define DCs, and show the proportion of cDCs, CD11b^+^ cDCs, CD103^+^ cDCs, and pDCs in MLNs of mature offspring of vehicle (Fig 3A) and TCDD-treated dams (Fig 3B). Prior to IAV infection, there were significantly fewer cDCs, CD11b^+^ cDCs in particular, in the MLN of offspring of the TCDD-treated dams (Fig 3C and 3D). One day after IAV infection, we observed a reduction in number of total cDCs, CD11b^+^ cDC and CD103^+^ cDC subsets, as well as pDCs (Fig 3C–3F). Three days after infection, the number of cDCs and pDCs remained significantly decreased in adult offspring that were developmentally exposed compared to those from control dams (Fig 3C–3F). We also observed a reduced percentage of CD103^+^ cDCs and increase in the proportion of CD11b^+^ cDCs one day after IAV infection, but these differences were not observed in uninfected offspring or 3 days after infection (S1 Table). In contrast to the MLN, in the lung there were no statistically significant differences in the percentage or number of cDCs or pDCs between treatment groups prior to or after infection (S1 Fig and S1 Table).

To determine whether the decreased number of DCs in the MLN was dependent on expression of AHR in CD11c^+^ cells, we used *Ahr*^*fx/fx*^*CD11c*^*cre*^ conditional knockout mice [38]. Specifically, *Ahr*^*fx/fx*^ dams, which express a functional AHR protein, were mated with male *Ahr*^*fx/fx*^*CD11c*^*cre*^ mice. Impregnated dams were dosed with 10 μg TCDD/kg body weight or vehicle control. This increased dose was used because *Ahr*^*fx/fx*^ mice express an allelic variant of the AHR, *Ahr*^*d/d*^, which encodes a protein with 10 times lower affinity for TCDD than the *Ahr*^*b/b*^ expressed by B6 mice [52]. Using adult *Ahr*^*fx/fx*^ and *Ahr*^*fx/fx*^*CD11c*^*cre*^ offspring of vehicle and TCDD-treated dams, we determined the number of DC subsets in the MLN 3 days after IAV infection (Fig 3G–3J). Maternal TCDD treatment significantly reduced number of all cDCs and of CD11b^+^cDCs and CD103^+^ cDCs, but not pDCs, in *Ahr*^*fx/fx*^ offspring, compared to *Ahr*^*fx/fx*^ offspring of vehicle treated dams (Fig 3G–3J). In contrast, this reduction was not observed in *Ahr*^*fx/fx*^*CD11c*^*cre*^ littermates that were developmentally exposed to TCDD (Fig 3G–3J). Additionally, while the number of cDCs appears diminished in vehicle control *Ahr*^*fx/fx*^*CD11c*^*cre*^ offspring compared to their vehicle *Ahr*^*fx/fx*^ littermates, the difference in these values was not statistically significant. Overall, these findings suggest that AHR activation during development decreases the number of DCs in the lung-draining lymph node via a mechanism that, at least in part, requires AHR expression in cells of the CD11c lineage.

### Developmental AHR activation reduces DC migration

**Fig 4:**
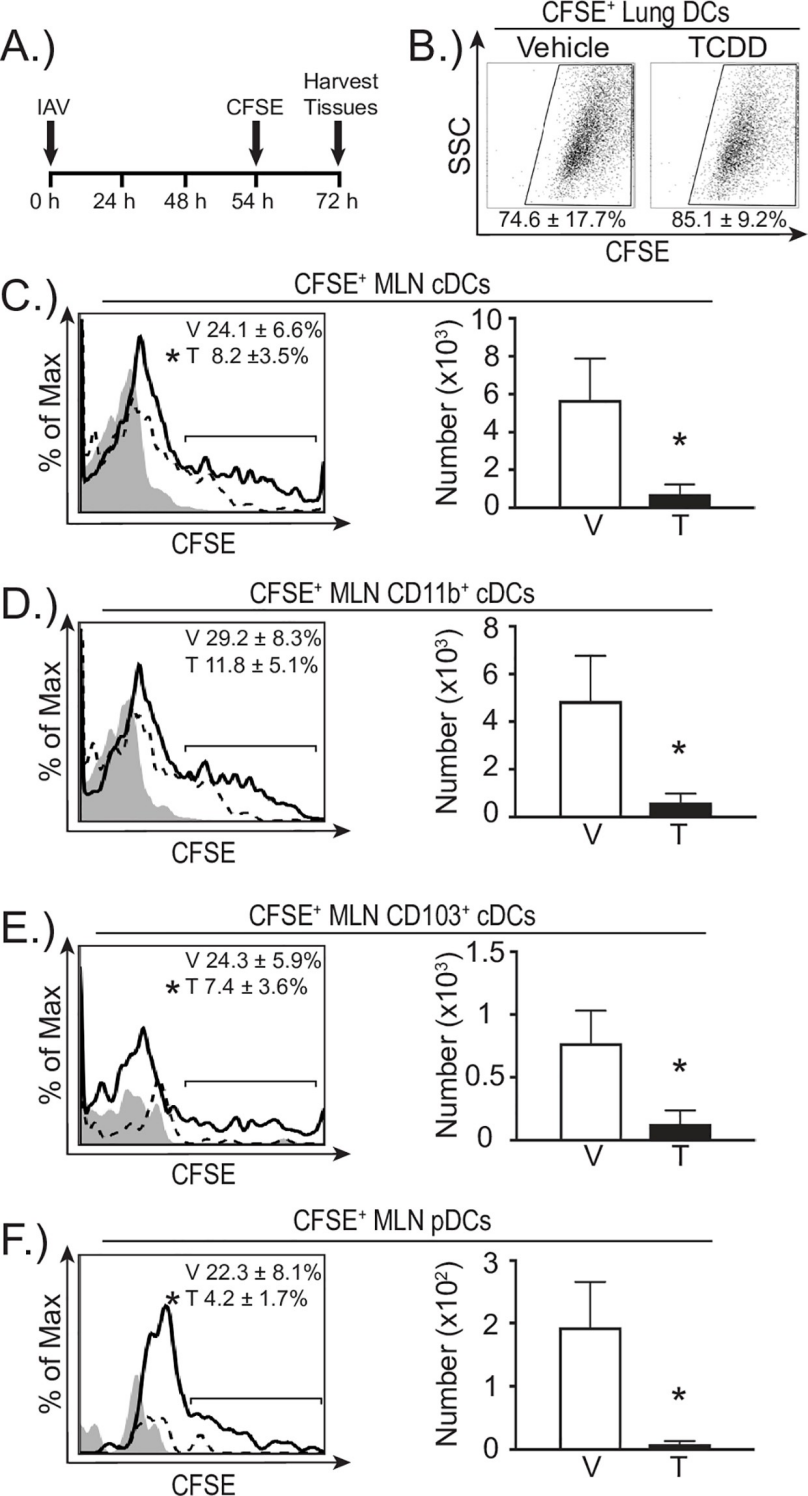
*In vivo* DC trafficking from the lung to MLN is reduced in offspring of TCDD treated dams. **(A)** At maturity, developmentally exposed offspring were infected with IAV (HKx31). CFSE (8mM) diluted in PBS was instilled (i.n.) 54 h after infection. Mice were sacrificed 18 h after CFSE treatment and cells were stained for flow cytometry. **(B)** Representative FACS plots show CFSE^+^ DCs in lungs of vehicle or TCDD exposed mice. The average percentage (±SEM) of DCs that were CFSE^+^ is shown below plots. **(C-F)** CFSE^+^ DCs in the MLN of TCDD (T) or vehicle (V) exposed adult offspring were enumerated 72 h after infection. Representative histograms show the CFSE^+^ staining of each DC subset (vehicle, solid line; TCDD, dotted line; gray depicts the CFSE FMO control). The average percentage (±SEM) of each DC subset that was CFSE^+^ is indicated on each plot. The bar graphs show the average number of the indicated DC subset that was CFSE^+^. Error bars depict ± SEM. An * indicates p ≤ 0.05. All offspring within a group are from a separate dam (n = 3–7 per group). Data are from one experiment that is representative of two independent experiments. Underlying data can be found in S1 Data.

The decreased number of DCs in the MLN of offspring that were developmentally exposed to TCDD could reflect that developmental exposure increases DC death and/or affects DC migration. Apoptotic and dead DCs were identified using flow cytometry. Regardless of whether we examined DCs prior to or after infection, we did not observe any statistically significant differences in apoptotic or dead DCs from the MLN or lungs of offspring from TCDD-treated dams compared to vehicle offspring (S2 Fig). In contrast, we found evidence that developmental exposure affects DC migration. To directly measure the ability of DCs to emigrate from the infected lung to the MLN, we fluorescently labeled cells in the respiratory tract with CFSE, and enumerated CFSE^+^ DCs in the MLN 3 days after infection [35, 53] (Fig 4A). The percentage of DCs in the lung that were CFSE^+^ was not different between offspring of dams treated with vehicle or TCDD (Fig 4B). Thus, developmental exposure did not affect the labeling of DCs by intranasally instilled CFSE. However, early life activation of the AHR reduced the percentage of CFSE^+^ DCs in the MLN compared to CFSE^+^DCs in MLNs of infected offspring of vehicle treated dams (Fig 4C–4F, histograms). Furthermore, this reduction spanned all DC subsets, and there were significantly fewer CFSE^+^ cDCs, CD11b^+^, CD103^+^, and pDCs in the MLN 3 days after infection in offspring of TCDD-treated dams (Fig 4C–4F, bar graphs). These findings indicate that AHR activation during development leads to DCs with a diminished capacity to emigrate from the lung to lymph node during infection.

**Fig 5:**
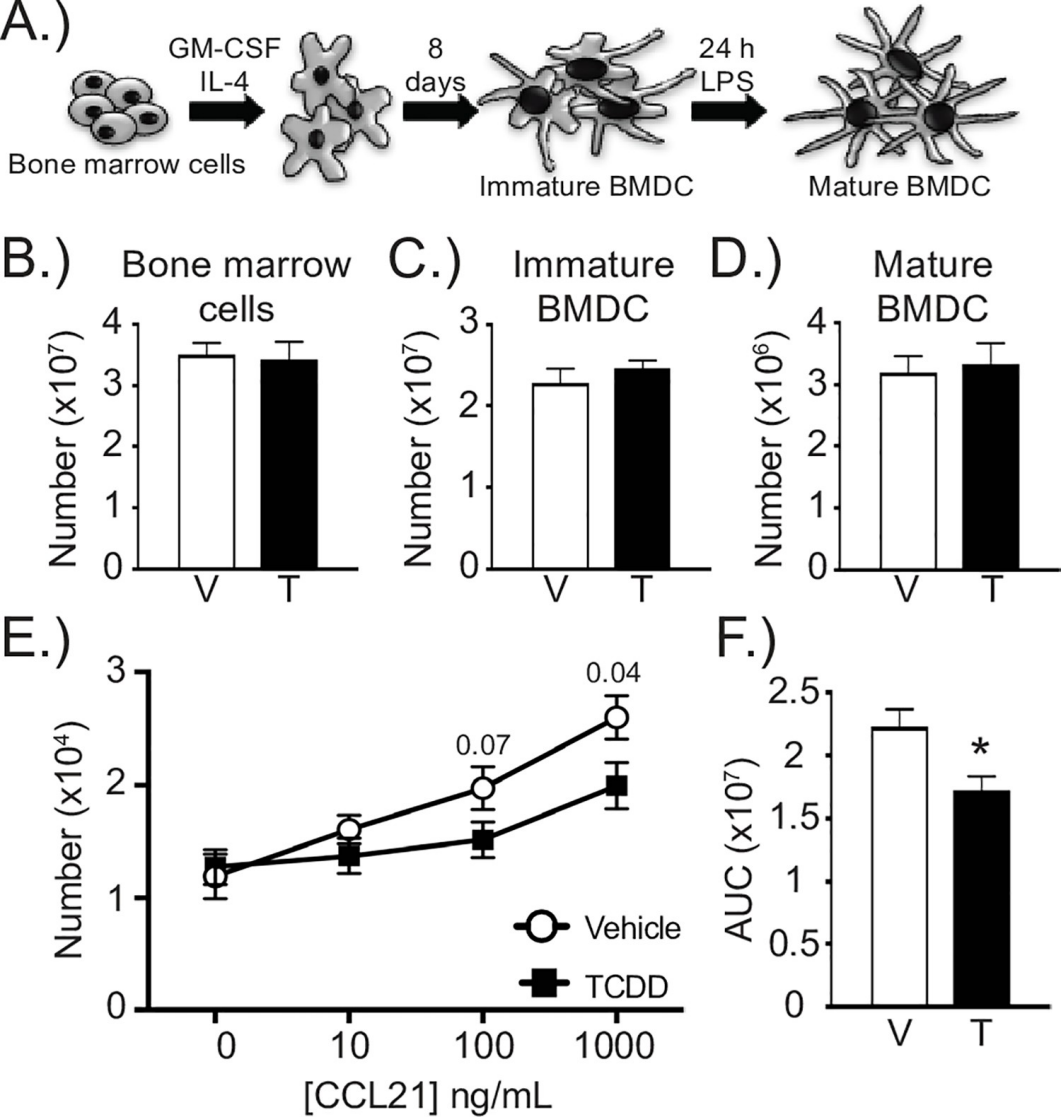
*In vitro* BMDC migration is reduced by developmental AHR activation. **(A)** Bone marrow dendritic cells (BMDC) were generated from bone marrow cells of uninfected offspring of vehicle and TCDD treated dams. **(B-D)** The bar graphs depict the number of bone marrow cells **(B)**, the number of immature BMDC **(C)**, and the number of mature CD11c^hi^MHCII^hi^ BMDCs **(D)** from adult offspring in each group. **(E, F)** Migration towards a gradient of CCL21 was determined using a Transwell system. **(E)** The graph depicts the average number of BMDCs collected from the bottom well at each CCL21 concentration. p-values are listed above data points. **(F)** The bar graph shows the average area under the curve (AUC) for DC migration in the two treatment groups. Error bars depict ± SEM. An * indicates p ≤ 0.05. BMDCs from all offspring within a group are from a separate dam (n = 12 mice per group). Data were combined from two independently conducted experiments. Underlying data can be found in S1 Data.

To further test whether developmental exposure blunts DC migration properties, we used a well-established *in vitro* DC migration assay. Specifically, we generated DCs from the bone marrow of naïve adult offspring of dams treated with either vehicle or TCDD (Fig 5A), and measured their ability to migrate towards a concentration gradient of CCL21 in a Transwell system. Consistent with prior reports, maternal treatment with this low dose of TCDD did not alter the total number of bone marrow cells obtained [29], nor did it affect the number of immature BMDCs generated after day 8 in culture, nor the number of mature BMDCs obtained following LPS treatment (Fig 5B–5D). However, fewer DCs from the TCDD-exposed group migrated from the upper toward the lower chamber, containing CCL21 (Fig 5E). Area under the curve (AUC) analyses further support that the proportion of migrated DCs was significantly reduced for BMDCs generated from animals that were developmentally exposed to TCDD (Fig 5F). These results further support that early life activation of the AHR results in DCs with a reduced ability to migrate towards an optimal stimulus.

**Fig 6:**
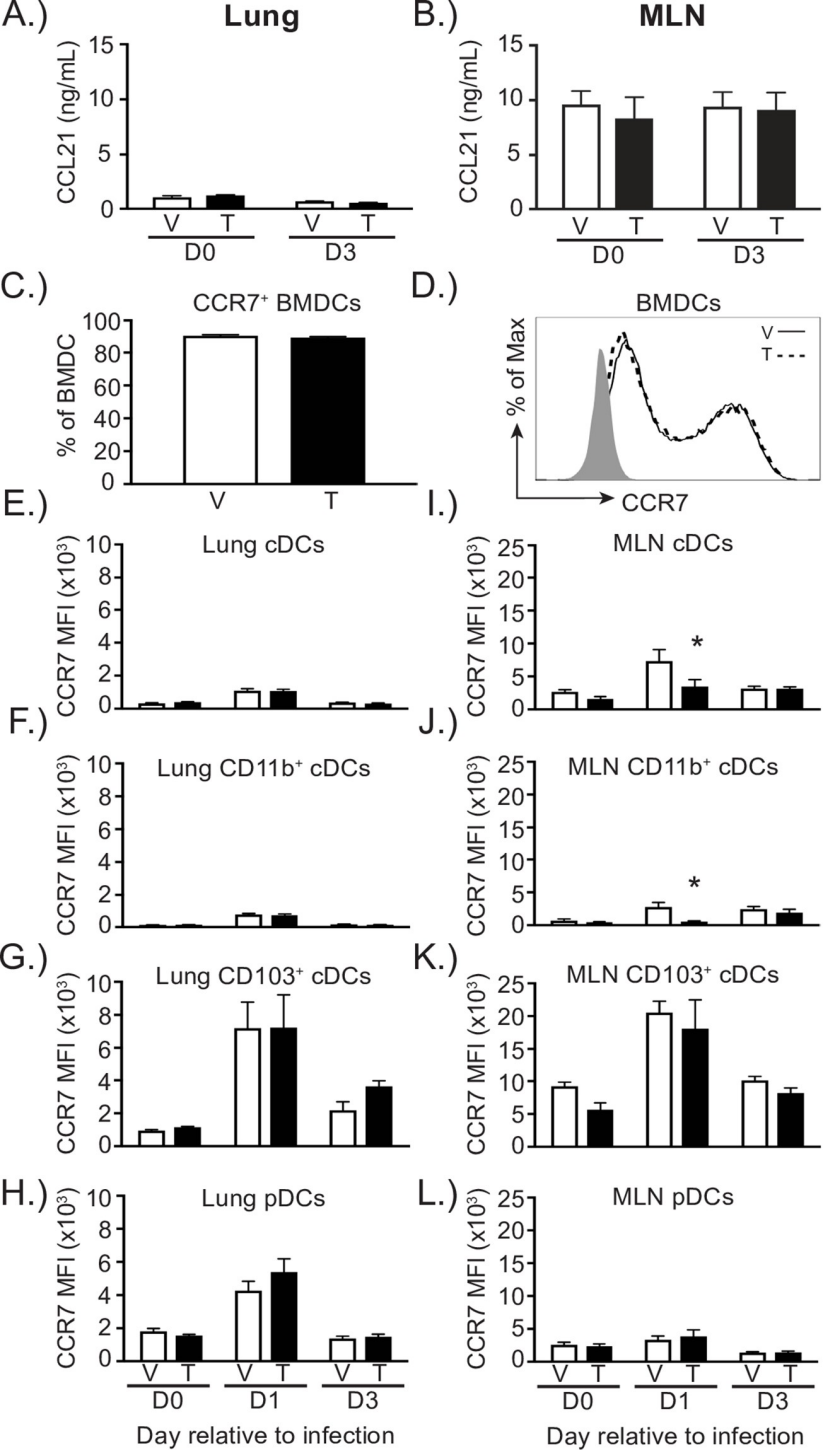
CCL21 production is not affected by early life activation of the AHR, while CCR7 levels are moderately changed on cDC in the MLN. (**A-B**) CCL21 levels in lung and MLN homogenates were measured by ELISA. The bar graph shows the levels of CCL21 in ng/mL from the lung (**A**) and MLN (**B**) of uninfected (d0) or infected (d3; HKx31) offspring. (**C-L**) Single cell suspensions from BMDC, lung, and MLN were stained for flow cytometric analysis, and DC subsets are defined as in Fig 3 with the addition of an anti-CCR7 antibody. **(C)** The bar graph shows the percentage of LPS stimulated BMDC that are CCR7^+^. **(D)** The histogram shows mean fluorescence intensity of CCR7 on either V (solid line) or T (dotted line) exposed LPS stimulated BMDCs. Solid histogram represents FMO control. (**E-H**) The bar graphs show the geometric mean fluorescence intensity (MFI) of CCR7 on DC subsets in the lung prior to (day 0) or after infection (HKx31; days 1–3). (**I-L**) The bar graphs show the MFI of CCR7 on the indicated DC subsets in the MLN prior to or after infection (HKx31; day 0 or days 1–3, respectively). Error bars depict ± SEM. An * indicates p ≤ 0.05. All offspring within a group are from a separate dam (n = 6–9 mice per group per day). Lung CCL21 data are representative of one experiment, and MLN CCL21 data are representative of two independent experiments. CCR7 data are from a single experiment that is representative of two independent experiments. Underlying data can be found in S1 Data.

Given that major regulators of leukocyte migration are CCL21 and chemokine receptor 7 (CCR7), the receptor that CCL21 binds [54–56], we next examined whether developmental exposure decreased *in vivo* levels of CCL21, or diminished CCR7 expression on DCs. Using ELISAs to measure CCL21 in tissue homogenates, the levels of CCL21 were about nine times higher in the MLN than the lung (Fig 6A and 6B). However, prior to and three days after infection, the levels of CCL21 in the lung and MLN were equivalent between developmental exposure groups (Fig 6A and 6B). Using BMDCs from developmentally exposed offspring, there was no difference in the percentage of BMDCs that were CCR7^+^ or in the mean fluorescence intensity (MFI) of CCR7 on BMDCs from these two exposure groups (Fig 6C and 6D). Similarly, in the lung the relative level of expression of CCR7 on DCs, examined using MFI, was not reduced on any of the DC subsets examined (Fig 6E–6H). Moreover, there were no differences in the percentage or number of CCR7-expressing DC subsets in the lung (S1 Table). In contrast, cDCs, specifically CD11b^+^ cDCs, in the MLN of developmentally exposed offspring had significantly reduced CCR7 levels 1 day after infection (Fig 6I–6L). In addition to, the proportion of all cDCs and of CD11b^+^ cDCs that were CCR7^+^ on day 1 after infection in the MLN was also reduced (S1 Table). However, this decrease in CCR7 expression was transient, as the MFI and percentage of CCR7^+^ DCs in the MLN was not different between the two groups prior to infection or 3 days after infection.

**Fig 7:**
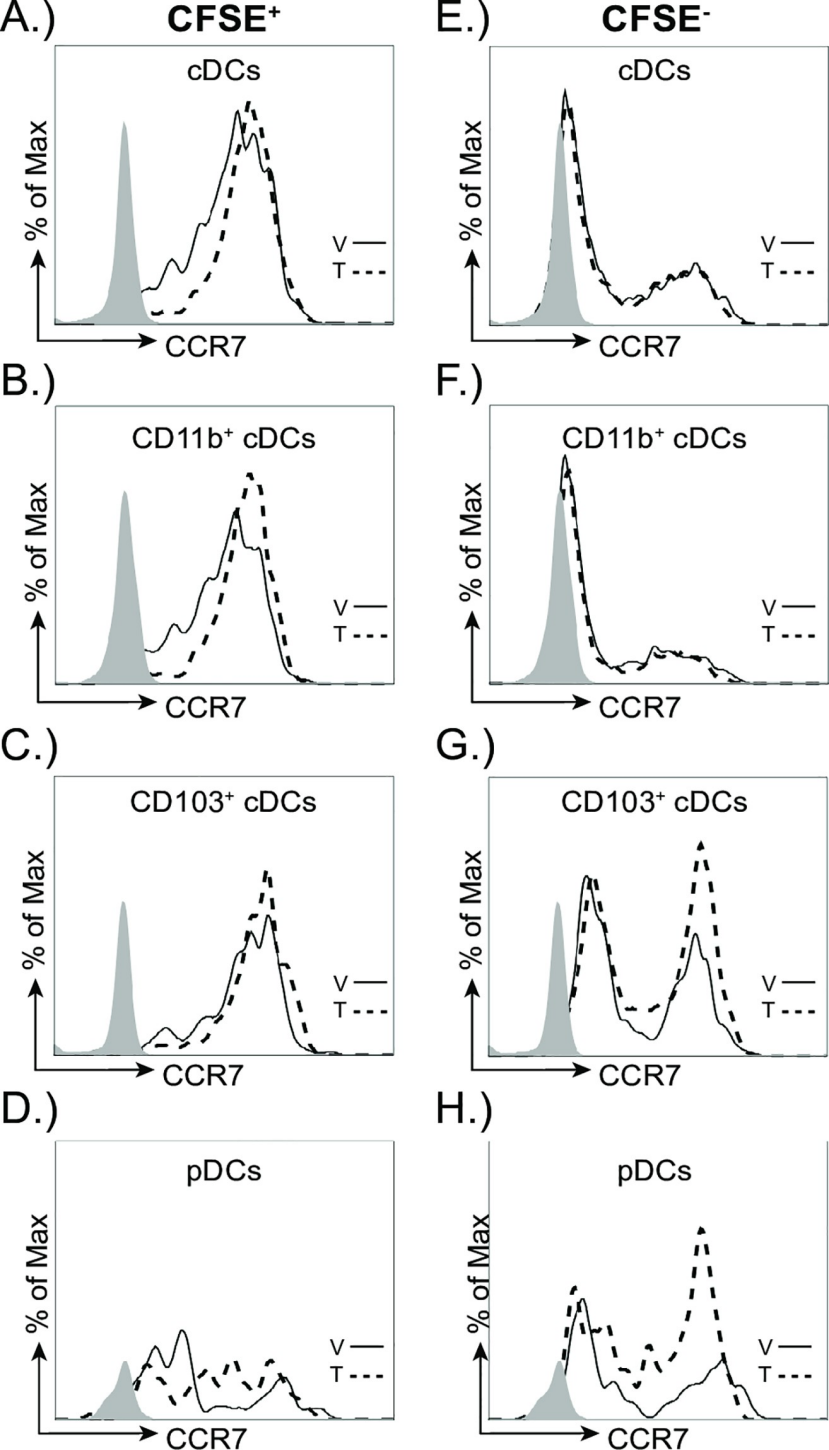
CCR7 levels on resident or recently migrated DC are not changed with early life exposure to TCDD in the MLN. At maturity, developmentally exposed offspring were infected with H3N2 IAV (HKx31). CFSE (8mM) was instilled (i.n.) 54 h after infection. Mice were sacrificed 18 h after CFSE treatment and single cell suspensions were made from MLN. Vehicle (V, solid line), TCDD (T, dotted line). Grey histograms indicate the fluorescence minus one (FMO) control. (**A-D**) Representative histograms depict CCR7 levels on recently migrated (CFSE^+^) DC subsets in the MLN 3 days after IAV infection. (**E-H**) Representative histograms depict CCR7 levels three days after infection on resident CFSEˉ DC subsets in MLNs of vehicle or TCDD exposed offspring. Error bars depict ± SEM. All offspring within a group are from a separate dam (n = 3–9 mice per group per day). Data are representative of at least two experiments with similar results.

To further determine whether developmental exposure affects CCR7 levels specifically on the DCs that trafficked to the MLN from the infected lung, we compared CCR7 levels on CFSE^+^ and CFSE^-^ DCs in the MLN (Fig 7). Overall, the expression of CCR7 was higher on CFSE^+^ cDC subsets (Fig 7A–7D) compared to CFSE^-^ cDCs (Fig 7E–7H). Also, a greater proportion of the CFSE^+^ cDCs were CCR7^+^, which further suggests that CCR7 is higher on cDCs in the MLN that emigrated from the lung. However, between the treatment groups, the MFI of CCR7 was not significantly different on any of the DC subsets examined (Table 1). These data suggest that early life AHR activation does not change CCR7 levels on DCs that have emigrated from the lung to the MLN.

**Table 1:** Mean fluorescence intensity (MFI) of CCR7.

DC type	CFSE^+^		CFSE^-^	
	Vehicle	TCDD	Vehicle	TCDD
cDCs	2710 ± 125	2784 ± 390	1029 ± 226	991 ± 96
CD11b^+^	2527 ± 242	2269 ± 468	857 ± 317	700 ± 78
CD103^+^	4531 ± 584	5516 ± 444	1899 ± 217	2474 ± 152
pDCs	1975 ± 1068	1893 ± 475	2186 ± 622	1692 ± 216

Table shows the MFI (± SEM) of CCR7 on each CFSE^+^ and CFSEˉ DC subset in the MLN three days after IAV infection. All offspring within a group are from a separate dam (n = 3–9 mice per group per day). Data are representative of at least two experiments with similar results.

### Developmental activation of AHR alters gene expression in DCs

**Fig 8:**
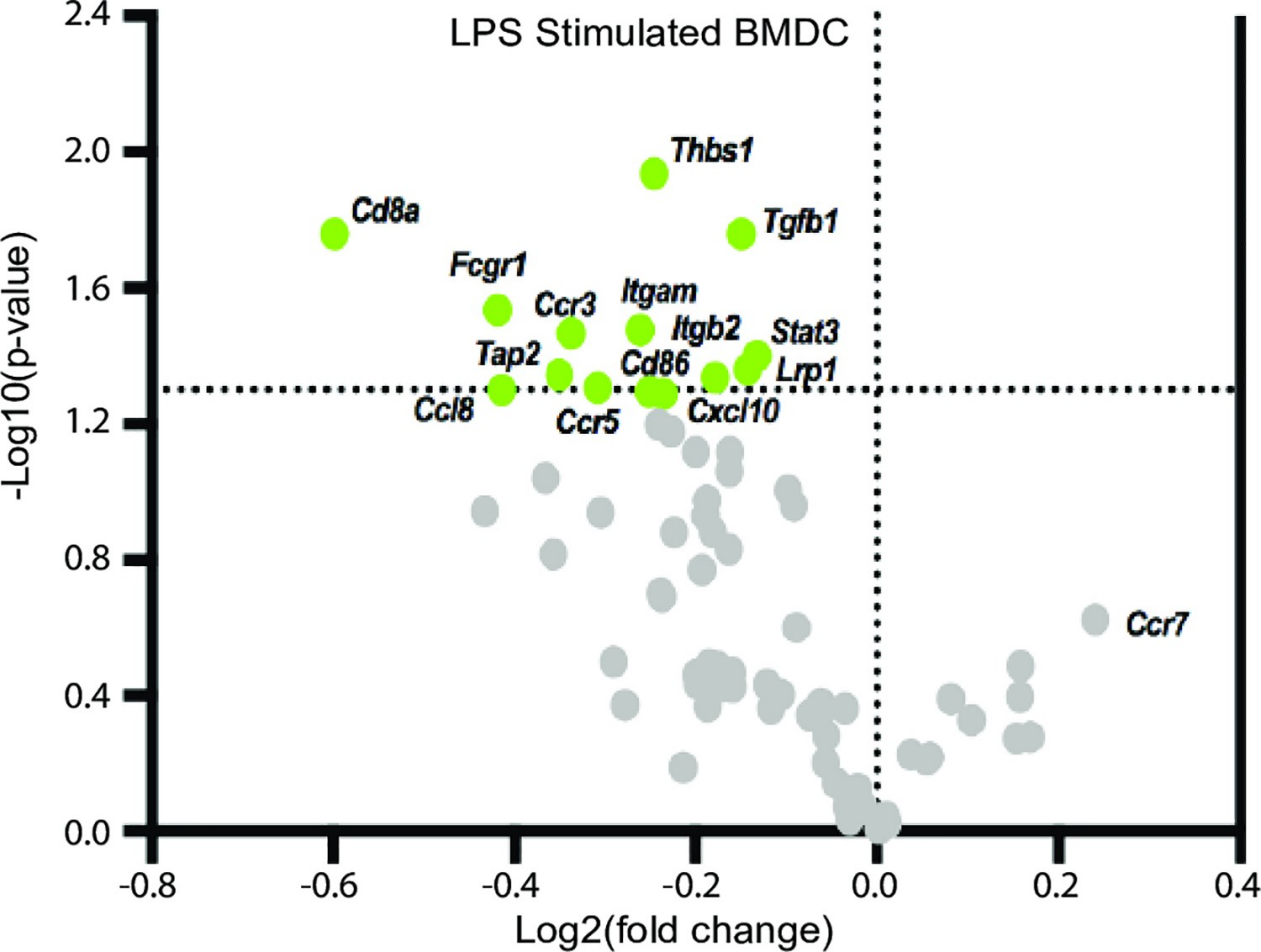
Developmental activation of the AHR down-regulates BMDC genes involved in pathways critical for DC function. Mature BMDC were generated from adult offspring (8–10 weeks old) of B6 dams treated with vehicle control or TCDD. The volcano plot shows -log10(p-value) vs. log2(fold change) of genes assayed by PCR array in LPS stimulated BMDC. Log2(fold change) calculated relative to LPS stimulated BMDCs from offspring developmentally exposed to vehicle. The horizontal dotted line indicates significance at -log10(0.05). Genes were considered significantly different when p ≤ 0.05. All offspring within a group are from a separate dam (n = 6 mice per group). Underlying data can be found in S1 Data.

Given that early life AHR activation reduced the capacity of DCs to stimulate naïve CD8^+^ T cells and also affected DC trafficking, we next wanted to identify DC-specific functional pathways that are altered by developmental AHR activation. To uncover cellular pathways in DCs affected by developmental exposure, we utilized a PCR array that measured 84 genes involved in DC function. In mature BMDCs from offspring of dams that were treated with vehicle or TCDD, 71 of 84 genes were expressed. Of these genes, 14 were differentially expressed in BMDCs from offspring of TCDD-treated dams (Fig 8), while the remaining 58 genes were not significantly different between the two exposure groups. Interestingly, all 14 of the differentially expressed genes (DEGs) were down-regulated in the TCDD group, compared to BMDCs from the control group (Table 2). The 14 DEGs encode proteins that contribute to five aspects of DC function: antigen processing and presentation, cellular adhesion and migration, chemokines, cellular survival and differentiation, and signal transduction.

**Table 2:** Significantly differentially expressed genes in BMDC.

Gene ID	^a^Log2(fold change)	^b^p-value	Pathway
CD8a	-0.60	0.02	Antigen processing and presentation
**FCGR1**	-0.42	0.03	Antigen processing and presentation
**LRP1**	-0.14	0.04	Antigen processing and presentation
TAP2	-0.35	0.05	Antigen processing and presentation
CD86	-0.24	0.05	Antigen processing and presentation
THBS1	-0.25	0.01	Cellular adhesion and migration
**ITGAM**	-0.26	0.03	Cellular adhesion and migration
CCR3	-0.34	0.03	Cellular adhesion and migration
ITGB2	-0.18	0.05	Cellular adhesion and migration
CCR5	-0.31	0.05	Cellular adhesion and migration
TGFB1	-0.15	0.02	Cellular survival, proliferation, and differentiation
CCL8	-0.41	0.05	Chemokines
CXCL10	-0.25	0.05	Chemokines
STAT3	-0.13	0.04	Signal transduction

The table shows the log2(fold change), p-value, and associated pathway of significantly differentially expressed genes in BMDC. Bolded genes represent genes that were similarly observed in MLN DC (Table 3). All offspring within a group are from a separate dam (n = 6 mice per group).

^a^Values calculated relative to Vehicle LPS BMDC

^b^All expression changes were statistically significant with p ≤ 0.05

**Fig 9:**
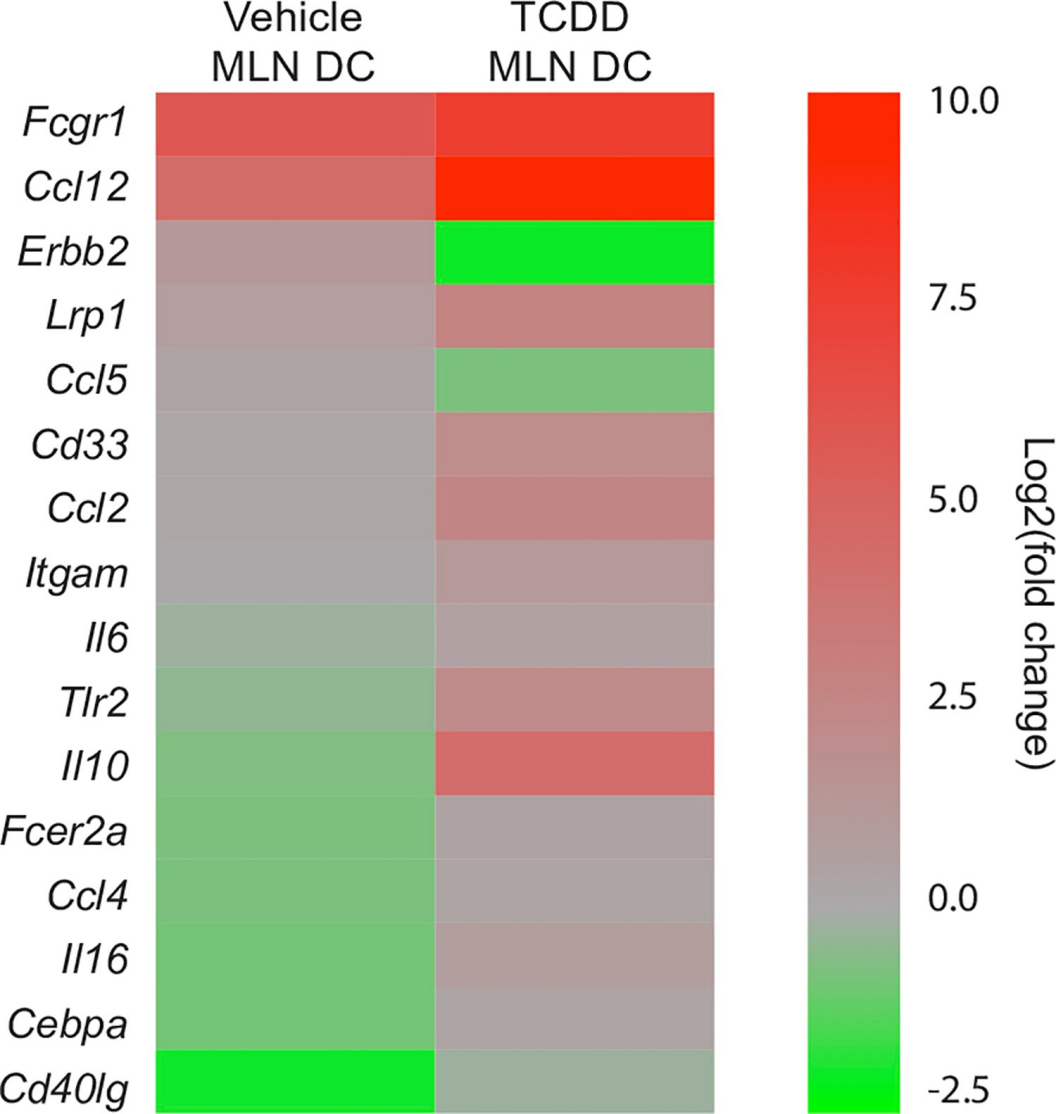
Genes in MLN DC are up-regulated after early life AHR activation. CD11c^+^ cells (DC) were enriched from the MLNs of adult offspring from dams that were exposed to vehicle control or TCDD. The heat map shows significantly differentially expressed genes (DEGs) in DC from vehicle or TCDD exposed offspring 3 days after IAV infection (HKx31) based on the average log2(fold change) relative to naïve DCs from 3 replicate samples per group and are ordered from most significantly up-regulated to most significantly down-regulated in the vehicle group. Genes were considered significantly different between vehicle and TCDD groups when p ≤ 0.05. All offspring within a group are from a separate dam (n = 12 mice per replicate, 3 independent replicates per group). Underlying data can be found in S1 Data.

Using the same PCR array system, we compared gene expression in DCs from MLN of developmentally exposed mice. In MLN-derived DCs, there were 16 DEGs, while 63 genes were not significantly different between the two exposure groups and 5 genes were not detected (Fig 9). The number of DEGs was similar in BMDCs and MLN-derived DCs. Yet, in contrast to DEGs in BMDCs, the direction of change among the DEGs from MLN-derived DCs was mostly increased (Fig 9). The differences in the change in direction may reflect differences in the timing of DC stimulation relative to gene expression assessment and differences in DC activation *in vitro* versus *in vivo*. Despite these differences, the DEGs in DCs from MLN were similar to BMDCs with regard to the functional pathways affected. Similar to BMDCs, pathways changed by developmental exposure include antigen processing and presentation, cellular adhesion and migration, cellular survival and differentiation, chemokines, cytokines, and signal transduction pathways (Table 3). Thus, although only three DEGs were the same in MLN DCs and BMDCs (*Lrp1*, *Fcgr1 and Itgam)*, the overall pathways affected by developmental exposure were similar in DCs from the MLN and bone marrow.

**Table 3:** Significantly differentially expressed genes in MLN DC.

Gene ID	^a^Log2(fold change) from naïve DCs		^b^p-value	Pathway
	^a^Vehicle	^a^TCDD		
TLR2	-0.43	2.09	0.00	Antigen processing and presentation
CD40lg	-1.96	-0.22	0.01	Antigen processing and presentation
FCER2a	-0.76	0.45	0.03	Antigen processing and presentation
**LRP1**	0.71	2.70	0.04	Antigen processing and presentation
**FCGR1**	5.83	7.58	0.04	Antigen processing and presentation
CD33	0.19	2.00	0.05	Antigen processing and presentation
**ITGAM**	0.09	1.11	0.02	Cellular adhesion and migration
CEBPa	-0.89	0.24	0.04	Cellular survival, proliferation, differentiation
CCL2	0.13	2.50	0.01	Chemokines
CCL12	4.31	9.31	0.01	Chemokines
CCL4	-0.78	0.26	0.02	Chemokines
CCL5	0.30	-0.77	0.03	Chemokines
IL6	-0.89	0.81	0.00	Cytokines
IL10	-0.70	4.27	0.01	Cytokines
IL16	-0.23	0.51	0.02	Cytokines
ERBB2	1.15	-2.02	0.00	Signal transduction

The table shows the log2(fold change) from naïve vehicle or TCDD DCs, p-value, and associated pathway of significant DEGs in DC. Bolded genes represent genes that were similarly observed in BMDC (Table 2).

^a^Values calculated relative to respective naïve MLN DC treatment group

^b^All expression changes were statistically significant with p ≤ 0.05

In addition to the genes in the PCR array, we measured *Ccr7*, *Ido1 and Cyp1a1* expression. There were no statistically significant differences in the expression of *Ccr7* in BMDCs or MLN DCs from the TCDD group compared to DCs from the vehicle exposed offspring (Fig 8 and S2 Table). This corresponds with the general lack of durable differences in CCR7 protein expression on DCs in offspring of vehicle and TCDD treated dams (Fig 6). *Cyp1a1* and *Ido1* represent two genes that are upregulated by direct exposure to AHR agonists. The expression levels of *Ido1* in BMDCs or MLN DCs were not different between treatment groups (S2 Table). Consistent with prior reports [7], we did not detect *Cyp1a1* expression in MLN DCs from either exposure group (S2 Table). This suggests that the DEGs in cells from adult offspring are not direct AHR target genes, but instead reflect altered transcriptional regulation in DCs that is triggered by AHR activation earlier in life.

## Discussion

AHR activation during development has a lasting impact on adaptive immune responses in the offspring, modulating T cell responses in several model systems, including viral infection. Yet, our understanding of how early life AHR activation specifically affects functional properties of many types of leukocytes is scant. For instance, although DCs are critical for the initiation of adaptive immunity, the consequences of AHR activation during development on DCs has not been established. Given that direct AHR signaling modifies DC function, it is logical to hypothesize that exposure to exogenous AHR ligands during development could affect this important leukocyte lineage later in life. In this report, we show that developmental activation of the AHR influences some aspects of DC function, such as their ability to stimulate naïve CD8^+^ T cells and their ability to migrate, while leaving other DC characteristics without perceptible alteration. Thus, inappropriate AHR signaling early in life can lead to changes to immune defense mechanisms via durable modifications to DCs.

One critical function of DCs is the stimulation of naïve T cells during an immune challenge. Despite their central role, only a handful of studies have examined whether DCs are sensitive to perturbation by early life exposures, and just a few of these directly assessed APC function [57–62]. For instance, DCs from allergen-naïve offspring of asthmatic mothers exhibited an increased ability to stimulate proliferation of CD4^+^ T cells *in vitro* [57]. In another study, developmental exposure of mice to the antibiotic neomycin resulted in a reduction in the ability of a mixture of macrophages and DCs to stimulate antigen-specific CD4^+^ and CD8^+^ T cells [61]. Thus, the early life environment can influence APC function in offspring. Our findings add to this knowledge base by showing that developmental exposure to an AHR ligand affects this important property of DCs.

Direct AHR activation influences the ability of DCs to activate naive T cells [34, 35, 38, 63, 64]. Therefore, it is not surprising that developmental exposure to an AHR ligand influences this central DC function. However, a notable difference between direct versus developmental exposure may be that developmental AHR activation leads to differential effects on the capacity of DCs to stimulate naïve CD4^+^ versus CD8^+^ T cells. Here, we show that in a head-to-head comparison, triggering AHR during development leads to DCs that have a reduced capacity to activate virus-specific naïve CD8^+^ T cells, but there were no differences in their ability to activate naïve CD4^+^ T cells during IAV infection. AHR activation during development, such as via maternal exposure to TCDD, disrupts activation of both naïve CD4^+^ and CD8^+^ T cells *in vivo* [7, 8]. The TCR transgenic T cells used to measure DC APC function were from untreated and uninfected mice, allowing us to interrogate the potential DC contribution to this altered T cell response. Two *in vivo* studies of direct AHR activation showed that DCs from TCDD-exposed mice were equally capable of stimulating OVA-specific CD4^+^ T cell responses, highlighting a difference between direct and developmental activation of AHR [33, 36]. However, activation of CD8^+^ T cells was not examined in these studies. Moreover, an impact on DC-CD4^+^ T cell activation has not been consistently observed with direct exposure. For example, in another study, splenic DCs from mice treated directly with TCDD showed enhanced proliferation of OVA-specific CD4^+^ T cells in an *ex vivo* assay [63]. In contrast, direct treatment of DCs with the AHR agonist, 2-(1′H-indole-3′-carbonyl)-thiazole-4-carboxylic acid methyl ester (ITE) reduced the proliferation of antigen-specific CD4^+^ T cells *in vitro* [34]. While less well studied in comparison to CD4^+^ T cells, direct activation of the AHR also affects their ability to stimulate CD8^+^ T cells. For example, fewer IAV-specific CD8^+^ T cells were activated by DCs isolated from TCDD treated mice [35, 38]. Thus, AHR signaling in the mature immune system, by direct treatment with an AHR ligand, affects the capacity of DCs to stimulate CD4^+^ and CD8^+^ T cells, although whether AHR activation enhances or diminishes T cell activation appears to be dependent on the context in which DCs were evaluated, while developmental AHR signaling affects the capacity of DCs to stimulate CD8+ T cells but not CD4^+^ T cells in the context of IAV infection.

While the mechanisms that contribute to the differential ability of DCs to activate CD4^+^ and CD8^+^ T cells following AHR activation during development are not yet known, our study provides important insight. For instance, this difference may reflect functional disparities among DC subsets, as distinct DC subsets (e.g., CD103^+^ vs CD11b^+^) may play a more dominant role in CD4^+^ vs. CD8^+^ T cell activation at distinct points in time after respiratory antigen challenge [38, 48–50]. Also, a unique function of some DC subsets, such as migratory CD103^+^ cDCs, is that they can present exogenously derived peptides with MHCI through a process called cross-presentation. Cross presentation is particularly important for initiating CD8^+^ T cell responses during viral infections and anti-tumor responses [65, 66]. There is some circumstantial evidence that direct (i.e. non-developmental) exposure to AHR agonists affects cross-presentation. Specifically, AHR activation reduced the ability of highly purified CD103^+^ DCs from IAV-infected mice to stimulate naïve virus-specific CD8^+^ T cells [38]. Further support that triggering AHR during development influences DC cross-presentation later in life comes from examination of gene expression in DCs from offspring of treated dams. DEGs in DCs include *Tap2*, *Fcgr1 and Lrp1*. *Tap2* encodes transporter associated with antigen processing 2 (TAP2), which is directly associated with cross-presentation by DCs [66]. *Fcgr1* encodes the high affinity immunoglobulin gamma Fc receptor I (FCGR1), which mediates internalization of antigen-IgG complexes and DC cross presentation [67, 68]. *Lrp1* encodes a scavenger receptor called low density lipoprotein receptor-related protein 1 or LRP1, which aids in the internalization of antigen-heat shock protein complexes [69–71]. Both FCGR1 and LRP1 play a role in cross-presentation of exogenous antigen in the context of MHCI on DCs [67–72]. Thus, disruption of cross-presentation is a mechanism by which early life activation of the AHR could dampen DC:CD8^+^ T cell interactions. Moreover, this provides a potential explanation for how AHR activation during development affects the ability of DCs to activate naïve CD8^+^, but not CD4^+^ T cells, following infection. In addition to cross presentation, inappropriate triggering of the AHR during development may also affect other mechanisms that govern DC and T cell interactions, such as the frequency and duration of DC:T cell contacts, antigen uptake, availability, and the balance between immunostimulatory and regulatory DC functions [73–76].

Another key finding is that AHR activation during development reduced DC migration later in life. Studies of direct AHR activation point to an emerging role for the AHR in the control of leukocyte migration. For example, direct exposure to TCDD reduced bone marrow cell migration *in vivo* [77]. In other studies, *in vivo* DC migration from the lung to the MLN during IAV infection was reduced upon AHR activation [35]. AHR signaling also affects neutrophil trafficking, although it may do so indirectly, via signaling in non-hematopoietic cells, and the direction of change depends upon the stimuli [30, 78–81]. Also, although AHR signaling affects neutrophil recruitment, it does not affect the number of neutrophils in peripheral tissues in the absence of antigen challenge [78–80]. Similarly, we did not observe differences in the number of DCs in lungs before or after infection. Yet, when cued to emigrate, such as *in vivo* during infection or *ex vivo* in response to a chemokine gradient, fewer DCs migrated. Although we observed fewer DCs emigrating from the lung to the MLN upon infection, it is possible that rather than failing to migrate, the DCs are migrating improperly and accumulating in a different anatomical site. Thus, while early life AHR activation influences DC migration properties, it likely does so in conjunction with, or in the context of, other signals to the DCs.

Although recent studies have linked direct AHR activation to increases in CCR7 expression levels [32, 82], developmental exposure led to only modest and transient decreases in CCR7 levels on cDCs in the MLN, and did not alter CCR7 expression levels on BMDCs or lung DCs. Thus, it is likely that other factors that regulate DC migration are affected by developmental AHR activation. For instance, signals that promote cellular cytoskeletal rearrangement or adhesion could be altered as a result of early life AHR activation in DC. Candidates revealed by gene expression analyses include molecular regulators of cell adhesion and migration, such as *Lrp1* and *Itgam* (CD11b). CD11b is one of four β_2_ integrins involved in cell signaling, migration, and in DC-T cell interactions [83]. In addition to playing a role in cross-presentation, LRP1 signaling can influence cell migration [84–87], and LRP1 directly associates with CD11b and modulates cellular adhesion [84, 88]. Interestingly, differential expression of *Lrp1* and *Itgam* was observed in both BMDCs and in DCs from the MLN of developmentally exposed mice. This further suggests a possible central role for AHR-mediated changes in these genes, or in the upstream regulators of the expression of these genes. An important consideration in the interpretation of gene expression analyses in DCs isolated from the MLN is that these results are skewed by preferentially reflecting gene expression in the DC populations that have already migrated to the lymph nodes. This limitation is mitigated by our observation that in developmentally exposed mice, decreased DCs number reflects a reduction in all subsets examined.

The changes observed in DCs appear to be inherent to the DC lineage, and suggest that triggering AHR during development causes long-lasting programming of DCs. Evidence includes the differences in *ex vivo* APC function, diminished DC migration, including abrogation of the reduced DC number in MLNs of developmentally exposed mice lacking *Ahr* in the CD11c lineage. Given that the lifecycle of DCs is several days to a few weeks [89–91], the DCs evaluated at maturity are not present in the fetus and neonate. That is, the DCs interrogated at maturity are not directly exposed to the exogenous AHR ligand given to the dams. Instead, it is likely that DC progenitor cells, such as a monocyte-dendritic cell progenitors (MDP) or common dendritic cell progenitors (CDP), are affected by early life AHR activation. Changes in DC function observed in developmentally exposed offspring may reflect alterations in epigenetic programming in the DC lineage. Epigenetic regulatory mechanisms, such as DNA methylation, influence gene expression and cellular function [92]. Although not extensively studied, developmental exposure to other environmental factors, such as being born to an asthmatic mother, alters DNA methylation marks in DCs of the offspring [57, 58]. Furthermore, early life activation of the AHR modifies DNA methylation patterns and gene expression in CD8^+^ T cells of adult offspring [7]. Thus, it is possible that inappropriate AHR activation during development alters DNA methylation, and potentially other epigenetic regulatory mechanisms, in the DC lineage, leading to durable changes in DC functions later in life.

The research reported here focuses on how developmental exposure to a representative AHR agonist influences the response of DCs during acute, primary IAV infection. DCs are fundamental to appropriate immune responses to many pathogens, to vaccines, and to establishing anti-tumor immunity. Thus, the significance of these initial findings extends beyond IAV infection, and suggest developmental exposure to AHR-binding substances affects DC responses in other contexts. More broadly, these data support the idea that the early life environment shapes DC function in an enduring manner, influencing immune function into adulthood. Given that DCs are critical regulators of adaptive immune responses during may different types of immune challenges, this work reveals that AHR-mediated events influence DCs may be an important factor in many communicable, and non-communicable, diseases.

## Materials and methods

### Mice

C57Bl/6 (B6) and B6.Cg-Tg (Itgax-cre) 1-1Reiz/J (CD11c^cre^) [93] mice (5–6 weeks old) were purchased from the National Cancer Institute (Frederick, MD) or The Jackson Laboratory (Bar Harbor, ME). Dr. Christopher Bradfield (University of Wisconsin, Madison, WI) provided breeding stock of *Ahr*^*fx/fx*^ mice. Breeding stock of F5 T cell receptor (TCR) transgenic mice (F5 mice) were provided by Dr. Demetrius Moskophidis (Medical College of Georgia, Augusta, GA) and Dr. Dimitris Kioussis (National Institute for Medical Research, London, UK). The TCR on CD8^+^ T cells of F5 mice recognizes amino acids 366–374 of the nucleoprotein (NP_366-374_) of influenza virus A/Memphis/102/72 in the context of H-2D^b^ [41]. Breeding stock of OTII TCR transgenic mice (OTII mice) were provided by Dr. Minsoo Kim (University of Rochester, Rochester, NY). The TCR on CD4^+^ T cells of OTII mice recognizes amino acids 323–339 of chicken ovalbumin in the context of I-A^b^ [94]. The phenotype of F5 and OTII mice is determined using flow cytometry [41, 94]. Conditional knockout *Ahr*^*fx/fx*^*CD11c*^*cre*^ mice were generated by mating male *CD11c*^*cre*^ mice with female *Ahr*^*fx/fx*^ mice. DNA from tail biopsies was obtained by digestion with Proteinase K (Invitrogen, Carlsbad, CA) and Direct PCR Lysis Reagent (Viagen Biotech, Los Angeles, CA). Genotyping was performed by PCR using the following primers: *Ahr*^*fx/fx*^ OL4064 (5’- CAG TGG GAA TAA GGC AAG AGT GA– 3’) and OL4088 (5’- GGT ACA AGT GCA CAT GCC TGC– 3’); CD11c *Cre* transgene forward (5’–ACT TGG CAG CTG TCT CCA AG– 3’) and CD11c *Cre* transgene reverse (5’–GCG AAC ATC TTC AGG TTC TG– 3’). All mice are housed in microisolator cages in a specific pathogen-free facility at the University of Rochester Medical Center, and are provided food and water ad libitum. Animals were sacrificed using anesthetic overdose, followed by a secondary method. All animal treatments were conducted with approval of Institutional Animal Care and Use Committee and Institutional Biosafety Committee of the University of Rochester.

### Developmental exposure

Nulliparous female B6 mice were housed with B6 males, and checked daily for the presence of a vaginal plug; designated day 0 of gestation (GD0). Impregnated B6 mice were singly housed and treated with 1 μg TCDD/kg body weight or the peanut oil vehicle control (Vehicle) by gavage on GD 0, 7, and 14 and 2 days after parturition (PND2). This dose of TCDD is not overtly toxic to the dam or pups [7, 8, 29, 30]. In experiments where *Ahr*^*fx/fx*^*CD11c*^*cre*^ mice were used, female *Ahr*^*fx/fx*^ mice were mated with male *Ahr*^*fx/fx*^*CD11c*^*cre*^ mice to generate *Ahr*^*fx*/fx^ and *Ahr*^*fx/fx*^*CD11c*^*cre*^ offspring. *Ahr*^*fx/fx*^ dams were dosed with 10 μg TCDD/kg body weight or vehicle control on GD14 and PND2. This increased dose was used because *Ahr*^*fx/fx*^ mice express an allelic variant of the AHR, *Ahr*^*d/d*^, which encodes a protein with 10 times lower affinity for TCDD than the *Ahr*^*b/b*^ expressed by B6 mice [52, 80, 95]. The timing of dosing of impregnated *Ahr*^*fx/fx*^ mice was altered due to pup death that occurred when TCDD was administered during early pregnancy (i.e., dams were not treated on GD0 and GD7). For the dosing solution, TCDD (≥99% purity; Cambridge Isotope Laboratories, Woburn, MA) was dissolved in anisole and diluted in peanut oil. The vehicle control consisted of peanut oil containing an equivalent concentration of anisole (0.01%). No culling of litters was performed, and offspring were weaned at 20–21 days of age.

### Viral infection

Adult offspring (8–12 weeks of age) of TCDD- or vehicle-treated dams were anesthetized by intraperitoneal (i.p.) injection of avertin (2,2,2-tribromoethanol; Sigma Aldrich, Milwaukee, WI) and inoculated intranasally (i.n.) with 25 μl sterile PBS containing 120 hemagglutinating units (HAU) of influenza virus strain A/HK/x31 (x31; H3N2), 1x10^7^ plaque forming units (PFU) of A/Memphis/102/72 (Mem/102; H3N2), or 8.25x10^5^ PFU of A/HK/x31/OVAII (x31/OVAII; H3N2; [44]). This work was conducted with prior review and approval of the Institutional Biosafety Committee of the University of Rochester.

### Isolation of immune cells

Single-cell suspensions of mediastinal lymph node (MLN), lung, bone marrow, and spleen cells were obtained as previously described [29, 35, 96]. Briefly, MLNs were disrupted between the frosted ends of 2 microscope slides, and digested with collagenase-containing media for 25 min at 37°C in 5% CO_2_ (RPMI 1640 medium containing 1 mg/mL collagenase A (Worthington Biochemical, Lakewood, NJ), 30 μg/mL DNase I (Roche), 2.5% FBS, and 10 mM HEPES). Lungs were perfused with a solution of 0.6 mM EDTA (Invitrogen, Carlsbad, CA) in 1X phosphate buffered saline (PBS; Lonza, Walkersville, MD). Immune cells from the airways and interstitial spaces of the lung were obtained by digesting lungs with RPMI containing collagenase and DNAse I. Bone marrow (BM) cells were collected from the long bones suspended in complete RPMI (cRPMI) media (RPMI 1640 containing 10% FBS, 100 U/mL penicillin/streptomycin, 2 mM Glutamax-I (L-alanyl-L-glutamine dipeptide), 1 mM sodium pyruvate, and 1X non-essential amino acids; Gibco, Grand Island, NY). Naïve F5 CD8^+^ T cells and naïve OTII CD4^+^ T cells were enriched from spleens of untreated and uninfected mice using the MagCelect Mouse Naïve CD8^+^ T Cell Isolation Kit, or MagCelect Mouse Naïve CD4^+^ T Cell Isolation Kit, respectively (R&D Systems, Minneapolis, MD). The purity of naïve CD8^+^ T cells (Vβ11^+^CD8^+^CD44^lo^) and naïve CD4^+^ T cells (Vβ5^+^CD4^+^CD44^lo^) was determined by flow cytometry, and was >95%.

### Flow cytometry

Cells were incubated with anti-mouse CD16/32, and stained with previously determined optimal concentrations of fluorochrome-conjugated mAbs. To identify DCs, antibodies against MHC class II (M5/114.15.2; I-A/I-E), CD11c (N418), CD11b (M1/70), CD103 (M290), B220 (RA2-6B2), PDCA-1 (ebio927). In some experiments, fluorochrome-conjugated antibodies for annexin V, live/dead, or CCR7 (4B12) were added. To identify T cells, antibodies against CD8 (53.67), Vβ11 (RR3–15), CD4 (GK1.5), Vβ5 (MR9-4), and CD44 (IM7) were used. Antibodies were purchased from eBioscience, BD Biosciences or BioLegend. Fluorescence minus one (FMO) controls were used to define gating parameters. Doublet discrimination and autofluorescent cell exclusion was included in the gating strategy. Data acquisition was performed using LSR-II cytometers (BD BioSciences) and data analyses were performed using FlowJo software (Tree Star, Ashland, OR).

### Ex vivo assay of APC function

DCs were isolated from pooled MLN of Mem/102- or X31/OVAII-infected adult offspring (>30 mice per group) and enriched using a Mouse CD11c Microbead Kit (Miltenyi Biotec, Auburn, CA) [35, 38]. On the same day, naïve T cells from spleens of F5 or OTII mice were isolated using MagCelect Mouse Naïve CD8^+^ T Cell Isolation Kit or Naïve CD4^+^ T Cell Isolation Kits, respectively (R&D Systems, Minneapolis, MD), and labeled with 2 μM carboxyfluorescein diacetate succinimidyl ester (CFSE; Invitrogen, Carlsbad, CA) [97]. CFSE-labeled naïve F5 CD8^+^ or CFSE-labeled OTII CD4^+^ T cells (2x10^5^) were cultured in 96-well plates with serially diluted CD11c^+^ MLN cells. After 3 (F5) or 4 (OTII) days in culture, cells were collected and stained with antibodies to Vβ11 and CD8, or Vβ5 and CD4, along with CD44 for flow cytometric analysis. Activated and proliferated T cells were defined based on up-regulation of CD44 and loss of CFSE staining (CFSE^decay^CD44^hi^) [38].

### Fluorescent immunohistochemistry

MLNs were snap frozen in OCT (Sakura Finetek, Netherlands). Using a cryostat, whole lymph nodes were cut into 10 μm thick sections, and 3–4 sections were placed onto coated slides. To determine the middle section, the total number of sections for each MLN was divided in half. The slide containing this section, and the slides with serial sections directly before and after it were stained, giving a total of 6–9 stained MLN sections per sample. Tissues were fixed with 2% paraformaldehyde (PFA), washed with PAB (1X phosphate buffer saline (PBS), containing 1% bovine serum albumin (BSA), and 0.1% sodium azide). Fixed slides were stained with a cocktail of the following fluorochrome-conjugated antibodies: CD11c PE (N418; 0.004 μg/μl; eBioscience), CD19 APC (1D3; 0.004 μg/μl; eBioscience), CD4 PerCP (RM4-5; 0.006 μg/μl; BD Biosciences), and LYVE-1 AF488 (ALY7; 0.01 μg/μl; eBioscience). An anti-mouse CD16/CD32 (93; eBioscience) antibody was included to reduce nonspecific binding to Fc receptors. Fluorescent images were captured using a conventional fluorescence microscope with an Olympus DP80 camera and automated stage (Olympus Corporation, Waltham, MA). Olympus cellSens software was used to create montage fluorescent images of each MLN. Image analysis was performed on raw images using an open source software distribution of ImageJ called Fiji (https://fiji.sc/) [98]. Determination of T cell zones and B cell zones was performed using Fiji’s polygon tool, by drawing demarcations based on anti-CD19 and anti-CD4 staining. Images were thresholded to determine the percentage of CD11c positive area across the entire surface area, and in each compartment.

### In vivo DC migration

CFSE was diluted in sterile endotoxin-free PBS to 8 mM. Mice were anesthetized and diluted CFSE was instilled (i.n.) 54 h after infection with IAV (HKx31), to label all cells in the respiratory tract [35, 53]. After 18 h of CFSE treatment (3 d after infection), mice were sacrificed and MLN cells were stained with antibodies against MHCII and CD11c to identify DCs that had migrated from the lung (CFSE^+^CD11c^+^MHCII^+^).

### BMDC generation and ex vivo migration assay

To generate bone marrow derived DCs (BMDCs), bone marrow cells (2x10^6^ cells/well) were cultured in cRPMI supplemented with 25 ng/mL mouse GM-CSF and 10 ng/mL mouse IL-4 (Peprotech, Rocky Hill, NJ) for 8 days [99, 100]. On day 8, immature BMDCs were harvested and resuspended in cRPMI, and treated for 24 h with LPS (1 μg/mL) to create mature DCs [99, 101]. After 24 h, mature BMDCs were enumerated, and used in migration assays, stained for flow cytometry, or had RNA extracted. Migration towards a gradient (0–1000 ng/mL) of CCL21 (Peprotech, Rocky Hill, NJ) was determined by plating 2x10^5^ BMDCs in the top wells of 8 μm pore, 96-well Transwell plates (Corning, Corning, NY) and enumerating cells in the bottom well after 2 h incubation at 37°C.

### Cytokine and chemokine analysis

Culture supernatants were collected from wells of DC:T cell co-cultures and the concentration of IFNγ was measured using a sandwich ELISA (BD Biosciences). CCL21 concentrations were measured in lung and MLN homogenates using a pre-fabricated CCL21 ELISA kit (R&D Systems, Minneapolis, MD) per the manufacturers protocol.

### Real-time PCR analysis

Total RNA was isolated from LPS stimulated BMDC with an RNeasy Mini Kit (Qiagen, Germantown, MD) and cDNA generated using iScript cDNA Synthesis Kit (Bio-Rad, Hercules, CA). RNA was isolated from CD11c^+^ DC enriched from MLNs using an RNeasy Plus Kit (Qiagen, Germantown, MD) and cDNA conversion was performed using a NuGEN WT-Ovation PicoSL Kit (NuGEN Technologies, San Carlos, CA). A mouse Dendritic and Antigen Presenting Cell RT^2^ Profiler PCR Array System was used (Qiagen, Germantown, MD; https://www.qiagen.com/us/shop/pcr/primer-sets/rt2-profiler-pcr-arrays/?catno=PAMM-406Z#geneglobe) following the manufacturer’s instructions. Additional gene-specific amplification was achieved using the following primers: mouse *Ccr7* (forward 5’GGA AAA TGA CAA GGA GAG CCA3’, reverse 5’GAG ACA AGA ACC AAA AGC ACAG3’; exon 1–2, IDT), mouse *Ido1* (forward 5’GCA TAA GAC AGA ATA GGA GGCA3’, reverse 5’GGT ACA TCA CCA TGG CGT AT3’; IDT), mouse *Cyp1a1* (forward 5’TTT GGA GCT GGG TTT GAC AC3’, reverse 5’CTG CCA ATC ACT GTG TCT A3’; IDT), and mouse *L13* as internal housekeeping gene (forward 5’CTA CAG TGA GAT ACC ACA CCA AG3’, reverse 5’TGG ACT TGT TTC GCC TCC TC’; IDT). Real-time RT PCR was performed using a Bio-Rad CFX96 Touch detection system with RT^2^ SYBR Green qPCR Master Mix (Qiagen, Germantown, MD) or iQ SYBR Green Supermix (Bio-Rad, Hercules, CA). Changes in gene expression were determined using the 2^-ΔΔC^_T_ method [102].

### Statistical analyses

The dam was defined as the statistical unit for all experiments, because the dams, not her offspring, were directly treated with TCDD or vehicle control. For most experiments, experimental groups were comprised of 6–9 adult male offspring from separate dams. For some *ex vivo* assays, pooling of mice from within the same exposure group was required to yield ample biological material. Statistical analyses were performed using JMP software (SAS Institute, Cary, NC). A two-way ANOVA, followed by a Tukey HSD post hoc test, was used to compare differences between multiple independent variables (e.g. multiple genotypes, over time, or across different concentrations). Differences between means of vehicle or TCDD groups at a single point in time were evaluated using a Student's *t*-test. Means were considered significantly different when p-values were less than or equal to 0.05. Error bars on all graphs represent the standard error of the mean (SEM).

## Supporting information

S1 Fig Developmental activation of AHR does not significantly affect lung DC number. DCs were evaluated prior to and up to 3 days after infection with IAV (HKx31). Flow cytometry was used to identify DC subsets as follows: conventional DCs (cDCs; CD11c^hi^MHCII^hi^ cells), CD11b^+^ cDCs (CD11c^hi^MHCII^hi^CD11b^+^CD103^-^ cells), CD103^+^ cDCs (CD11c^hi^MHCII^hi^CD103^+^CD11b^-^ cells), and plasmacytoid DCs (pDCs; CD11c^lo^MHCII^hi^ PDCA1^+^CD45R^+^ cells). (A, B) Representative dot plots depict the gating used to define cDCs, CD11b^+^ cDCs, CD103^+^ cDCs and pDCs in the lungs of vehicle (V) and TCDD (T) exposed offspring after gating to exclude doublets, dead cells, and autofluorescent cells. The percentage on the plot indicates the average percentage of the indicated DC subset 3 days after infection. cDC and pDC percentages are of all immune cells in the lung, whereas CD11b^+^ DCs and CD103^+^ DCs indicate the proportion of CD11c^hi^MHCII^hi^ cells (cDCs). (C-F) The bar graphs show the number (±SEM) of the indicated DC population in the lung from naïve (day 0) or infected mice. At each point in time, all offspring within a group were from a separate dam, n = 6–9 mice per group per day. Day 0 data are representative of 4 independent experiments, day 1 data are representative of 3 independent experiments, day 3 data are representative of 6 independent experiments with similar results. Underlying data can be found in S1 Data.
(DOCX)

S2 Fig Early life activation of AHR does not increase DC death in lung or MLN. DCs were evaluated prior to and 3 days after infection with IAV (HKx31). Flow cytometry was used to identify DC subsets with the addition of annexin V and live/dead stains to detect apoptotic and dead cells. Specifically, annexin V binds phosphatidyl serine residues on the outer leaflet of exposed plasma membranes and live/dead covalently binds intracellular amines from cells with compromised membranes; the detection of cells double positive for these markers indicate dead cells. The bar graphs show the number (±SEM) of DC subsets that were double positive for Annexin V^+^LiveDead^+^ in the lung (A-C) and MLN (D-F) from naïve (day 0) or infected mice (day 3). At each point in time, all offspring within a group were from a separate dam, n = 6–9 mice per group per day. Underlying data can be found in S1 Data.
(DOCX)

S1 Table Percentage and number of DCs in lung and MLN of developmentally exposed offspring. Flow cytometry was used to identify DC subsets prior to and up to 3 days after infection with IAV (HKx31) as follows: conventional DCs (cDCs; CD11c^hi^ MHCII^hi^ cells), CD11b^+^ cDCs (CD11c^hi^MHCII^hi^ CD11b^+^CD103^-^ cells), CD103^+^ cDCs (CD11c^hi^MHCII^hi^ CD103^+^CD11b^-^ cells), and plasmacytoid DCs (pDCs; CD11c^lo^MHCII^hi^ PDCA1^+^CD45R^+^ cells). In separate experiments, cells were further defined by expression of CCR7. Previous gating excluded doublets and autofluorescent cells. Percentage and number of DC subsets are indicated in the table. ^a^Percentage of all immune cells in the lung. ^b^Percentage of MLN cells. ^c^Percentage of cDCs. For CCR7^+^ DC subsets, percentages are of cDC, CD11b^+^, CD103^+^, or pDC that were positive for CCR7. For CCR7^+^ DC subsets, all numbers are x10^3^. All values ± SEM. An * indicates significance compared to vehicle (p ≤ 0.05).
(DOCX)

S2 Table Fold change gene expression in DCs from developmentally exposed offspring. Mature BMDC were generated from bone marrow of naïve or DCs were enriched from the MLNs of IAV infected adult offspring from dams that were exposed to vehicle control or TCDD. The table shows the fold change of *Ccr7*, *Ido1*, and *Cyp1a1* in DCs relative to their respective vehicle (BMDC) or uninfected (MLN DC) controls. Changes in gene expression were determined using the 2^-ΔΔC^_T_ method. All offspring within a group are from a separate dam (BMDC, n = 6 mice per group; MLN DC, n = 12 mice per replicate, 3 replicates per group).
(DOCX)

S1 Data Underlying data for data figures and supplemental figures. (XLSX)

## Abbreviations

AHR: aryl hydrocarbon receptor
APC: antigen presenting cell
BM: bone marrow
BMDC: bone marrow derived dendritic cell
CCL: C-C motif chemokine ligand
CCL12: C-C motif chemokine ligand 12
CCL2: C-C motif chemokine ligand 2
CCL4: C-C motif chemokine ligand 4
CCL5: C-C motif chemokine ligand 5
CCL8: C-C motif chemokine ligand 8
CCR: C-C motif chemokine receptor
CCR3: C-C motif chemokine receptor 3
CCR5: C-C motif chemokine receptor 5
CCR7: chemokine receptor 7
CD: cluster of differentiation
CD33: cluster of differentiation 33
CD40lg: cluster of differentiation 40 ligand
CD86: cluster of differentiation 86
CD8a: cluster of differentiation 8a
cDC: conventional dendritic cell
CDP: common dendritic cell progenitor
CEBPa: CCAAT/enhancer-binding protein alpha
CFSE: carboxyfluorescein diacetate succinimidyl ester
cKO: conditional knockout
CTL: cytotoxic T lymphocyte
CXCL10: C-X-C motif chemokine 10
DC: dendritic cell
DEG: differentially expressed gene
ELISA: enzyme-linked immunosorbent assay
ERBB2: erb-b2 receptor tyrosine kinase 2
FCER2a: Fc epsilon receptor 2 alpha
FCGR1: immunoglobulin gamma Fc receptor I
FMO: fluorescence minus one
GD: gestational day
HAU: hemagglutinating unit
IAV: influenza A virus
IFNγ: interferon gamma
IL10: interleukin 10
IL16: interleukin 16
IL6: interleukin 6
ITGAM: integrin alpha M
ITGB2: integrin subunit beta 2
LN: lymph node
LPS: lipopolysaccharide
LRP1: low density lipoprotein receptor-related protein 1
MDP: monocyte-dendritic cell progenitor
MFI: mean fluorescence intensity
MLN: mediastinal lymph node
PCB: polychlorinated biphenyl
pDC: plasmacytoid dendritic cell
PFU: plaque forming unit
PND: postnatal day
SEM: standard error of the mean
STAT3: signal transducer and activator of transcription 3
TAP2: transporter associated with antigen processing 2
TCDD: 2,3,7,8-tetrachlorodibenzo-*p*-dioxin
TCR: T cell receptor
TGFB1: transforming growth factor beta 1
THBS1: thrombospondin 1
TLR2: toll-like receptor 2
