# Supplementary material for: Environmental cues received during development shape dendritic cell responses later in life
Source: PLoS One. 2018 Nov 9;13(11):e0207007. doi: 10.1371/journal.pone.0207007 (PMC6226176; doi:10.1371/journal.pone.0207007)
Supplement: S1 Fig — DCs were evaluated prior to and up to 3 days after infection with IAV (HKx31). Flow cytometry was used to identify DC subsets as follows: conventional DCs (cDCs; CD11chiMHCIIhi cells), CD11b+ cDCs (CD11chiMHCIIhiCD11b+CD103- cells), CD103+ cDCs (CD11chiMHCIIhiCD103+CD11b- cells), and plasmacytoid DCs (pDCs; CD11cloMHCIIhi PDCA1+CD45R+ cells). (A, B) Representative dot plots depict the gating used to define cDCs, CD11b+ cDCs, CD103+ cDCs and pDCs in the lungs of vehicle (V) and TCDD (T) exposed offspring after gating to exclude doublets, dead cells, and autofluorescent cells. The percentage on the plot indicates the average percentage of the indicated DC subset 3 days after infection. cDC and pDC percentages are of all immune cells in the lung, whereas CD11b+ DCs and CD103+ DCs indicate the proportion of CD11chiMHCIIhi cells (cDCs). (C-F) The bar graphs show the number (±SEM) of the indicated DC population in the lung from naïve (day 0) or infected mice. At each point in time, all offspring within a group were from a separate dam, n = 6–9 mice per group per day. Day 0 data are representative of 4 independent experiments, day 1 data are representative of 3 independent experiments, day 3 data are representative of 6 independent experiments with similar results. Underlying data can be found in S1 Data. (DOCX) [file pone.0207007.s001.docx]

**S1 Fig.** Developmental activation of AHR does not significantly affect lung DC number
