# Supplementary material for: Environmental cues received during development shape dendritic cell responses later in life
Source: PLoS One. 2018 Nov 9;13(11):e0207007. doi: 10.1371/journal.pone.0207007 (PMC6226176; doi:10.1371/journal.pone.0207007)
Supplement: S2 Fig — DCs were evaluated prior to and 3 days after infection with IAV (HKx31). Flow cytometry was used to identify DC subsets with the addition of annexin V and live/dead stains to detect apoptotic and dead cells. Specifically, annexin V binds phosphatidyl serine residues on the outer leaflet of exposed plasma membranes and live/dead covalently binds intracellular amines from cells with compromised membranes; the detection of cells double positive for these markers indicate dead cells. The bar graphs show the number (±SEM) of DC subsets that were double positive for Annexin V+LiveDead+ in the lung (A-C) and MLN (D-F) from naïve (day 0) or infected mice (day 3). At each point in time, all offspring within a group were from a separate dam, n = 6–9 mice per group per day. Underlying data can be found in S1 Data. (DOCX) [file pone.0207007.s002.docx]

**S2 Fig.** Early life activation of AHR does not increase DC death in lung or MLN
