# Supplementary material for: Environmental cues received during development shape dendritic cell responses later in life
Source: PLoS One. 2018 Nov 9;13(11):e0207007. doi: 10.1371/journal.pone.0207007 (PMC6226176; doi:10.1371/journal.pone.0207007)
Supplement: S1 Table — Flow cytometry was used to identify DC subsets prior to and up to 3 days after infection with IAV (HKx31) as follows: conventional DCs (cDCs; CD11chi MHCIIhi cells), CD11b+ cDCs (CD11chiMHCIIhi CD11b+CD103- cells), CD103+ cDCs (CD11chiMHCIIhi CD103+CD11b- cells), and plasmacytoid DCs (pDCs; CD11cloMHCIIhi PDCA1+CD45R+ cells). In separate experiments, cells were further defined by expression of CCR7. Previous gating excluded doublets and autofluorescent cells. Percentage and number of DC subsets are indicated in the table. aPercentage of all immune cells in the lung. bPercentage of MLN cells. cPercentage of cDCs. For CCR7+ DC subsets, percentages are of cDC, CD11b+, CD103+, or pDC that were positive for CCR7. For CCR7+ DC subsets, all numbers are x103. All values ± SEM. An * indicates significance compared to vehicle (p ≤ 0.05). (DOCX) [file pone.0207007.s003.docx]

**S1 Table.** Percentage and number of DCs in lung and MLN of developmentally exposed offspring

| Lung | | | | | | | | | | | | |
| --- | --- | --- | --- | --- | --- | --- | --- | --- | --- | --- | --- | --- |
| Day p.i |  | ^a^cDCs | |  | ^c^CD11b^+^ | |  | ^c^CD103^+^ | |  | ^a^pDCs | |
|  |  | Veh | TCDD |  | Veh | TCDD |  | Veh | TCDD |  | Veh | TCDD |
| 0 | Percentage | 0.53 ± 0.14 | 0.56 ± 0.12 |  | 53.99 ± 2.74 | 60.32 ± 1.43 |  | 15.50 ± 1.10 | 13.21 ± 1.01 |  | 0.02 ± 0.006 | 0.02 ± 0.004 |
| 1 | Percentage | 0.73 ± 0.14 | 0.48 ± 0.13 |  | 56.52 ± 2.73 | 61.29 ± 3.58 |  | 15.47 ± 1.59 | 13.87 ± 1.87 |  | 0.02 ± 0.005 | 0.02 ± 0.006 |
| 3 | Percentage | 0.79 ± 0.16 | 1.14 ± 0.23 |  | 72.22 ± 4.43 | 78.65 ± 5.38 |  | 8.49 ± 1.48 | 6.58 ± 2.07 |  | 0.04 ± 0.010 | 0.06 ± 0.012 |
|  |  | CCR7^+^ cDCs | |  | CCR7^+^ CD11b^+^ | |  | CCR7^+^ CD103^+^ | |  | CCR7^+^ pDCs | |
| 0 | Percentage | 27.5 ± 1.23 | 24.8 ± 1.27 |  | 32.9 ± 1.59 | 27.4 ± 1.31* |  | 36.8 ± 1.11 | 39.8 ± 1.02 |  | 85.0 ± 2.42 | 85.4 ± 2.17 |
|  | Number x10^3^ | 9.86 ± 1.70 | 7.59 ± 0.92 |  | 6.15 ± 0.76 | 5.07 ± 0.61 |  | 2.05 ± 0.42 | 1.61 ± 0.21 |  | 1.22 ± 0.11 | 0.94 ± 0.13 |
| 1 | Percentage | 38.6 ± 4.82 | 28.9 ± 4.43 |  | 38.6 ± 4.31 | 29.8 ± 4.02 |  | 61.4 ± 7.14 | 45.8 ± 5.63 |  | 71.7 ± 2.89 | 77.8 ± 4.19 |
|  | Number x10^3^ | 25.39 ± 8.90 | 10.08 ± 3.78 |  | 13.40 ± 4.16 | 5.91 ± 1.90 |  | 6.62 ± 2.38 | 2.39 ± 1.01 |  | 1.40 ± 0.36 | 0.86 ± 0.23 |
| 3 | Percentage | 34.4 ± 3.65 | 34.6 ± 4.24 |  | 38.6 ± 4.02 | 39.5 ± 4.39 |  | 56.2 ± 4.38 | 66.1 ± 3.92 |  | 75.1 ± 3.14 | 83.3 ± 2.50 |
|  | Number x10^3^ | 19.76 ± 4.32 | 22.15 ± 4.52 |  | 16.27 ± 3.56 | 21.16 ± 4.75 |  | 2.51 ± 0.55 | 2.10 ± 0.20 |  | 2.07 ± 0.48 | 2.59 ± 0.51 |
| MLN | | | | | | | | | | | | |
| Day p.i |  | ^b^cDCs | |  | ^c^CD11b^+^ | |  | ^c^CD103^+^ | |  | ^b^pDCs | |
|  |  | Veh | TCDD |  | Veh | TCDD |  | Veh | TCDD |  | Veh | TCDD |
| 0 | Percentage | 0.30 ± 0.03 | 0.20 ± 0.05 |  | 52.55 ± 1.94 | 50.41 ± 3.65 |  | 17.24 ± 1.97 | 18.81 ± 3.92 |  | 0.06 ± 0.004 | 0.05 ± 0.005 |
| 1 | Percentage | 0.36 ± 0.07 | 0.18 ± 0.04 |  | 51.83 ± 2.95 | 67.30 ± 4.17* |  | 26.29 ± 2.87 | 16.53 ± 3.17* |  | 0.04 ± 0.010 | 0.04 ± 0.009 |
| 3 | Percentage | 0.34 ± 0.03 | 0.26 ± 0.05 |  | 56.58 ± 1.80 | 50.64 ± 3.61 |  | 16.56 ± 1.02 | 23.21 ± 3.36 |  | 0.07 ± 0.006 | 0.05 ± 0.005 |
|  |  | CCR7^+^ cDCs | |  | CCR7^+^ CD11b^+^ | |  | CCR7^+^ CD103^+^ | |  | CCR7^+^ pDCs | |
| 0 | Percentage | 53.37 ± 8.53 | 47.92 ± 5.93 |  | 46.80 ± 7.50 | 43.11 ± 4.95 |  | 92.31 ± 2.37 | 95.21 ± 1.05 |  | 84.72 ± 3.64 | 89.49 ± 0.92 |
|  | Number x10^3^ | 2.69 ± 1.19 | 2.69 ± 0.98 |  | 1.09 ± 0.40 | 1.84 ± 0.76 |  | 1.03 ± 0.50 | 0.60 ± 0.27 |  | 0.44 ± 0.14 | 0.88 ± 0.35 |
| 1 | Percentage | 81.67 ± 2.19 | 54.21 ± 4.01* |  | 75.38 ± 3.44 | 45.68 ± 3.58* |  | 97.75 ± 0.92 | 95.90 ± 1.14 |  | 74.22 ± 3.99 | 83.80 ± 1.62 |
|  | Number x10^3^ | 9.67 ± 3.47 | 1.04 ± 0.29* |  | 4.30 ± 1.36 | 0.57 ± 0.14* |  | 3.22 ± 1.46 | 0.32 ± 0.11 |  | 0.75 ± 0.16 | 0.34 ± 0.13 |
| 3 | Percentage | 83.14 ± 2.10 | 73.47 ± 7.48 |  | 77.36 ± 3.63 | 72.36 ± 7.86 |  | 95.67 ± 0.59 | 91.14 ± 4.64 |  | 70.52 ± 3.49 | 72.89 ± 2.89 |
|  | Number x10^3^ | 14.57 ± 2.38 | 4.83 ± 1.81* |  | 7.76 ± 1.31 | 2.89 ± 1.00* |  | 4.05 ± 0.61 | 1.15 ± 0.46* |  | 2.74 ± 0.82 | 0.73 ± 0.15* |
