# Supplementary material for: Environmental cues received during development shape dendritic cell responses later in life
Source: PLoS One. 2018 Nov 9;13(11):e0207007. doi: 10.1371/journal.pone.0207007 (PMC6226176; doi:10.1371/journal.pone.0207007)
Supplement: S2 Table — Mature BMDC were generated from bone marrow of naïve or DCs were enriched from the MLNs of IAV infected adult offspring from dams that were exposed to vehicle control or TCDD. The table shows the fold change of Ccr7, Ido1, and Cyp1a1 in DCs relative to their respective vehicle (BMDC) or uninfected (MLN DC) controls. Changes in gene expression were determined using the 2-ΔΔCT method. All offspring within a group are from a separate dam (BMDC, n = 6 mice per group; MLN DC, n = 12 mice per replicate, 3 replicates per group). (DOCX) [file pone.0207007.s004.docx]

**S2 Table.** Fold change gene expression in DCs from developmentally exposed offspring

| Gene |  | BMDC | |  | MLN DCs | |
| --- | --- | --- | --- | --- | --- | --- |
|  |  | Veh | TCDD |  | Veh | TCDD |
| *Ccr7* |  | 1.09 ± 0.21 | 1.14 ± 0.1 |  | 1.67 ± 0.62 | 0.51 ± 0.15 |
| *Ido1* |  | 1.1 ± 0.21 | 0.98 ± 0.07 |  | 0.60 ± 0.07 | 304.2 ± 299.4 |
| *Cyp1a1* |  | Not Evaluated | Not Evaluated |  | Not Detected | Not Detected |
